# In vitro propagation and morphological responses of a winter-hardy fern (*Phegopteris connectilis* (Michx.) Watt) under varying strength of MS medium and vitamin supplementation

**DOI:** 10.1186/s12870-026-09611-6

**Published:** 2026-07-28

**Authors:** Kanchan Soneji, Katarzyna Wróblewska, Renata Galek, Antoni Szumny, Jarosław Szadorski

**Affiliations:** 1 Department of Horticulture, Wrocław University of Environmental and Life Sciences, Wrocław, 50-375 Poland; 2 Department of Genetics, Plant Breeding and Seed Production, Wrocław University of Environmental and Life Sciences, Wrocław, 50-375 Poland; 3 Department of Food Chemistry and Biocatalysis, Wrocław University of Environmental and Life Sciences, Wrocław, 50-375 Poland; 4 Institute of Soil Science, Plant Nutrition and Environmental Protection, Wrocław University of Environmental and Life Sciences, Wrocław, 50-363 Poland

**Keywords:** Apogamy, Gametophyte, sporophyte induction, Thelypteridaceae, Beech fern, in vitro culture, MS medium

## Abstract

**Background:**

*Phegopteris connectilis* is a perennial fern distributed throughout the Northern Hemisphere, including Eurasia and North America. Although *Phegopteris connectilis* is widely distributed, local populations occurring in the Western Suwałki Lakeland region of North Eastern Poland are considered critically endangered, highlighting the need for efficient propagation methods to support conservation efforts. Because this species reproduces apogamously, the development of a reliable in vitro propagation system is important for both conservation and research. This study aimed to determine optimal Murashige and Skoog medium (MS) strength (MS, 1/2MS, 1/4MS and 1/8MS) combined with no, half, or full concentrations of vitamins for sporophyte induction.

**Results:**

Gametophytes exhibited a filamentous morphology, and sporophytes were induced within 6 weeks of culture. Different strengths of MS and vitamin formulations significantly affected growth parameters, including biomass accumulation, surface area, chlorophyll and carotenoid content. During stage-I, MS supplemented with vitamins (MS + V) enhanced sporophyte induction, whereas half-strength MS with half-strength vitamins (1/2MS + 1/2V) resulted in increase in biomass and gametophyte size. All analyzed nutrients, except Na, showed very strong positive correlations with sporophyte production, and all relationships were highly significant. In stage 2, transferring gametophytes from initial treatments to MS + V, gametophytes previously (in stage-I) cultured on MS demonstrated the highest multiplication factor. Notably, culturing gametophytes on MS medium for 10 weeks followed by transfer to MS + V enhanced sporophyte formation and increase on gametophyte by 2.29-fold. Additionally, gametophytes from 1/4MS and 1/8MS suggested a possible compensatory growth response after transferring on MS + V in stage-II.

**Conclusions:**

These findings demonstrate that nutrient composition strongly influences apomictic sporophyte induction and provide an efficient, reproducible protocol for sporophyte induction in *P. connectilis*, supporting its conservation and potential biotechnological applications.

**Supplementary Information:**

The online version contains supplementary material available at https://doi.org/10.1186/s12870-026-09611-6.

## Introduction

*Phegopteris connectilis* (Michx.) Watt (Thelypteridaceae) is a perennial winter hardy fern commonly known as Beech fern, widely distributed in northern hemisphere spanning Eurasia and North America [1, 2]. It is an apogamous fern commonly found in low temperature, shady, moist forests in marshy areas [3]. The rhizome is short spreading with fibrous roots, stipe is greenish-brown covered with fine hair. Fronds are compound, with bipinnate veins, serrated edges, tapered tips and bases, with sori on underside of frond surface arranged in parallel rows [4]. *Phegopteris connectilis* is grown as an ornamental fern indoors in India, used in residential gardens in Tennessee, and incorporated into urban green spaces, recreational areas, and social gathering places in Northern Kosovo and Metohija [5–7]. Local populations of *Phegopteris connectilis* occurring in the Western Suwałki Lakeland region of North Eastern Poland are designated as critically endangered, highlighting the need for conservation efforts [8]. Therefore, developing efficient propagation methods may provide a valuable complementary approach to support conservation efforts aimed at preserving these vulnerable local populations.

Ferns have been widely cultured in vitro using spores or explants (croziers, fronds, rhizome). Although there has been limited research on the in vitro cultivation of *Phegopteris connectilis*, a study of Soare et al., [9] has reported successful production of gametophytes and sporophytes through green sporangia (sori) and suggested apogamy as a characteristic feature of *Phegopteris connectilis* gametophytes. During amphimixis, sporogenesis primarily occurs within sporangium, undergoes meiosis, and produces haploid spores. On the contrary, in apomixis, the diplospores possessing a full chromosome set are capable of germinating [10]. Therefore, sporophyte emerges without fertilization (apogamy). This phenomenon has been observed in in-vitro cultures of *Dryopteris affinis* (Lowe) Fraser-Jenk., *Pteris tripartita* Sw., *Pityrogramma calomelanos* L. with green sporangia, spore derived gametophytes, juvenile leaves and croziers respectively used as an explant [11–13]. When considering apogamy, it is imperative to understand what causes this phenomenon. Initially, studies on *Cyrtomium falcatum* (L.f.) C.Presl and *Ceratopteris richardii* Brongn. showed positive influence of sucrose on induction of apogamous sporophytes [14, 15]. Over the past decade, studies in higher plants have demonstrated that in some lineages apomixis genes are highly methylated [10, 16]. Since, DNA methylation/demethylation can be driven by abiotic stressors and passed to the next generation under certain conditions, it is plausible to hypothesize that epigenetic modifications may underlie the transgenerational manifestation of apomixis in response to environmental cues [10, 17, 18]. This hypothesis is consistent with the findings in *Phegopteris polypodioides* and *Actiniopteris radiata* (Sw.) Link., wherein, nutrient limitation has demonstrated a pronounced effect on induction of apogamy [19, 20]. Apart from the culture media composition the main factors influencing apogamy in ferns include environmental stressors such as, water availability and light conditions as well as genetic regulation (induced and obligate apogamy) [21–23]. In previous studies on *Phegopteris connectilis*, Knop’s liquid medium was successfully used for in vitro culture [9, 24]. Owing to its low salt concentration, Knop’s medium is particularly suitable for spore germination and early gametophyte development, as it helps minimize osmotic stress [25]. On the contrary, Murashige & Skoog (MS) medium favors biomass accumulation, and sporophyte induction due to its high nutrient availability, which supports the higher energy demands of the developing sporophyte [26, 27]. Moreover, variations in results observed across different strengths of MS media are linked to the nutrient availability which vary depending on plant species and developmental stage [26]. Furthermore, vitamins act as cofactors in various biological processes.

Additionally, this work made use of gametophytes for culture initiation. Gametophytes as an explant provide numerous advantages as they are the free-living, haploid life stage preceding sporophyte formation. Unlike seed plants, ferns have an independent gametophyte phase that allows for unique conservation strategies. Cryopreservation of *Asplenium cuneifolium* Viv. gametophytes achieved 64–100% viability depending on cryopreservation method [28].

This study aims to investigate the effects of different strengths of Murashige and Skoog (MS) medium and vitamin supplementation on sporophyte induction, and to correlate the nutrient content of gametophytes with sporophyte development. It also evaluates associated physiological responses, including changes in chlorophyll content, carotenoid levels, and biomass growth, under varying MS medium concentrations. Furthermore, the study aims to identify the optimal media combinations for efficient sporophyte induction and sustained gametophyte multiplication through repeated subculture.

## Materials and methods

### In vitro spore culture

Clusters of *Phegopteris connectilis* gametophytes obtained from spores were used in the experiment. Fresh spores of *Phegopteris connectilis* were collected in August 2024 from Wrocław Botanical Garden, Wrocław, Poland with permission. The spores were rinsed in 70% ethanol and sterilized with 0.1% sodium hypochlorite for 20 min followed by rinsing with sterilized distilled water 5 times. To obtain gametophytes, sterilized spores were sown in MS medium [29] with 3% sucrose (Chempur, Poland), solidified with 1% (w/v) agar (CAS No. 9002-18-0, Chempur, Poland) and adjusted to pH-6.2 prior to addition of solidifying agent. Spores were kept in phytotron (SMART PRO KK75220002, POL-EKO A. Polok-Kowalska sp.k., Wodzisław Śląski, Poland) at 23 °C/21°C day/night temperature, with a 16/8 hour photoperiod at 50% relative air humidity. PPFD was 48–52 µmol m^−2^s^− 1^. Following sterilization, no visible contamination was observed during culture establishment and maintenance. Gametophytic mass that formed from germinated spores was further subcultured twice for 60 days on MS medium to obtain desired biomass. This gametophytic tissue was further used to study the effect of different combinations of MS medium strengths and vitamin supplementation on sporophyte induction (Stage 1) and the subsequent development and increase in the number of sporophytes on gametophytes following in vitro transfer to fresh culture media (Stage 2).

### Stage-1: sporophyte induction on gametophyte

To induce sporophytes, 4 pieces of 50 mg gametophytic tissue obtained after subculture were placed in petri dishes of Ø 5 cm with 12 different media combinations with varying strength of MS medium in presence (full)/absence/half of the required dose of vitamins (V). A vitamin solution (containing nicotinic acid 0.5 mg L^− 1^, pyridoxine 0.5 mg L^− 1^, and thiamine hydrochloride 1 mg L^− 1^) was added at a concentration of 1 mL L⁻¹.The combinations included: MS, MS + V, MS + 1/2V, 1/2MS, 1/2MS + V, 1/2MS + 1/2V, 1/4MS, 1/4MS + V, 1/4MS + 1/2V, 1/8MS, 1/8MS + V, 1/8MS + 1/2V, each with 3% sucrose and solidified with 1% (w/v) agar-agar adjusted to pH-6.2 prior to addition of solidifying agent. An experiment was established in 5 replications, one petri dish with 4 explants served as a replication. The plates were kept in phytotron at 23 °C/21°C day/night temperature, with a 16/8 hour photoperiod at 50% relative air humidity and PPFD 48–52 µmol m^− 2^ s^− 1^. Gametophytes were maintained in the different treatments until necrosis (browning due to cell death) was observed in the 1/4 MS and 1/8 MS treatments, which marked the endpoint of the stage-1. Sporophytes emerged from week 6 onward, and their number was recorded until week 10 and the induction rate (sporophyte/week) and specific sporophyte induction rate (sporophytes mg^− 1^ of gametophyte week^− 1^) was calculated (Table 1). The sporophyte induction rate was determined as the weekly increase in the average number of sporophytes during the induction period (weeks 6–10) and was calculated for each replicate as follows:

- **Sporophyte induction rate** = (number of sporophytes at week 10 − number of sporophytes at week 6) / 4.

  

The specific sporophyte induction rate was also calculated for each replicate as follows:

- **Specific sporophyte induction rate** = (number of sporophytes at week 10 − number of sporophytes at week 4) / (4 × 50), where 4 represents the duration of the induction period (weeks) and 50 represents the initial gametophyte biomass (mg) used for each treatment.

**Fig. 1:**
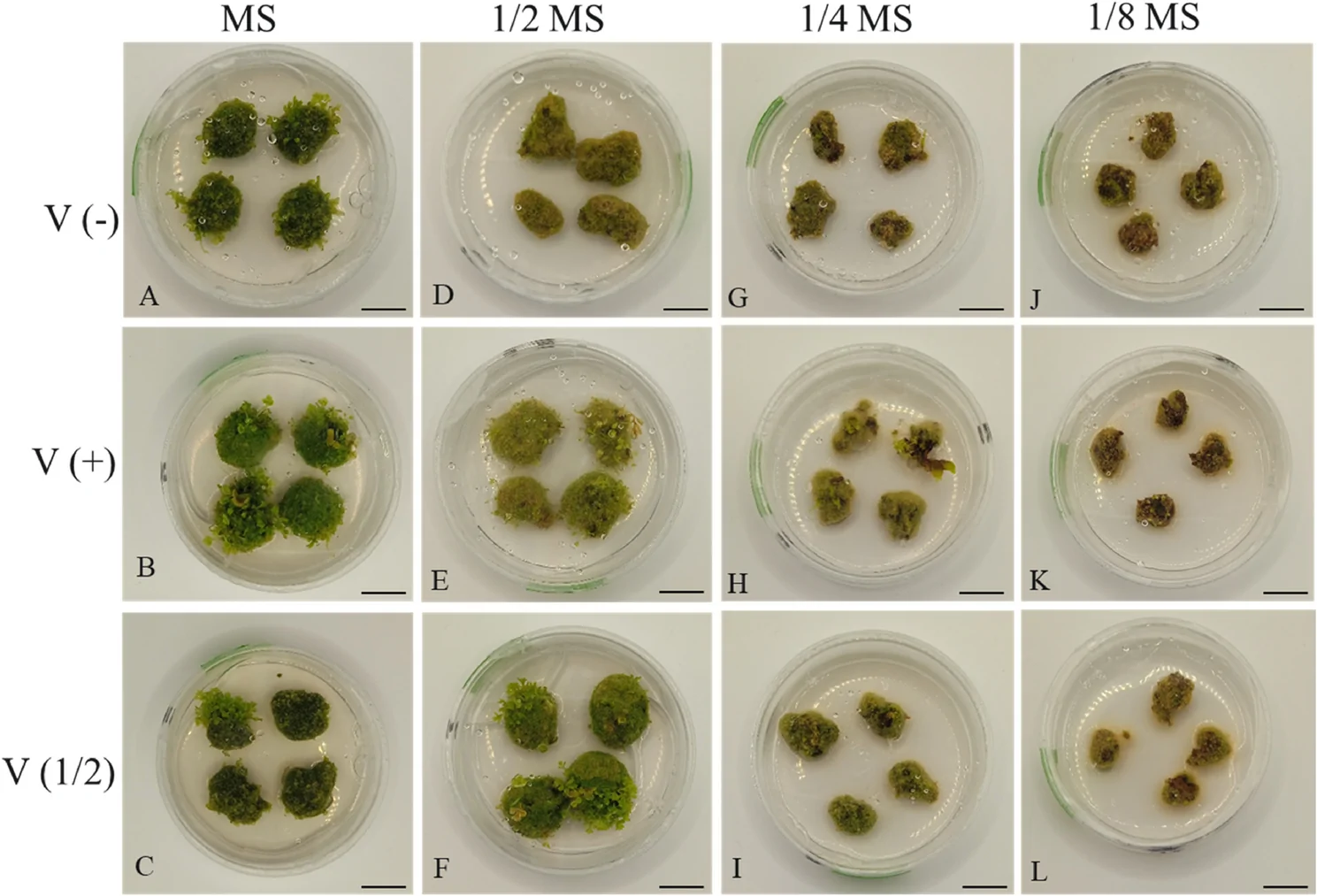
Effect of different MS medium and vitamin concentrations on the growth of *Phegopteris connectilis* gametophytes and induction of sporophytes at the end of stage-I. **A**–**L** Scale bar = 1 cm. MS – Murashige and Skoog medium; V – vitamins

On week 10, combinations 1/4MS and 1/8MS showed sign of necrosis (Fig. 1) after which half of the gametophyte from each treatment was transferred to stage-2 and other half was analysed for changes in size (mm^2^) using image J software [30], biomass (g), chlorophyll a & b (mg/g), carotenoid (µg/g) and nutrient concentration (mg/100 g dried weight) to assess the physiological status of gametophytes.

#### Photosynthetic pigment content analysis

0.4 g of fresh gametophyte was used to determine the photosynthetic pigment content. The chlorophyll a and b and carotenoid concentrations were measured at a density of 80% (v/v) acetone extracts using a spectrophotometer (type LPG422.99.00001, HACH LANGE GmbH, Düsseldorf, Germany) at wavelengths of 646, 663, and 470 nm. Analysis was carried out in 3 replicates. To calculate the concentrations of individual levels of photosynthetic pigments, the following formulas based on Porra [31] for chlorophyll a & b (mg/g) and Lichtenthaler & Wellburn [32] for carotenoids were used as mentioned below.

$$\text{Chl a}=12.2\times\mathrm{A663}-2.79\times\mathrm{A646}$$

$$\text{Chl b}=21.50\times\mathrm{A646}-5.10\times\mathrm{A663}$$

$$\mathrm{Carot}.=\left(1000\times\mathrm{A470}-1.82\times\text{Chl a}-85.02\times\text{Chl b}\right)/198$$

#### Nutrient content analysis

0.5 g of oven dried gametophyte was used to determine the concentration of macro-&micro-nutrients (P, K, Ca, Mg, Na, Zn, Fe, Mn, Cu and B) using microwave plasma–atomic emission spectrometer (4210 MP-AES, Agilent Technologies, Santa Clara, CA, USA) after mineralization in 65% nitric acid and 98% sulfuric acid using the Mars 6 mineralizer and post-digestion oxidation with 65% hydrogen peroxide. Analysis was carried out in 3 replicates. N analysis was carried out through macroelemental analyser (Vario Macro Cube, Elementar Analysensysteme GmbH, Langenselbold, Germany).

### Stage-2: subsequent development and increase in the number of sporophytes on gametophytes following in vitro transfer to fresh culture media

In this stage, MS + V medium was used as the reference control medium because it showed highest mean number of sporophytes during Stage I. Gametophytes from all treatments were transferred to this medium under 50 µmol m⁻² s⁻¹ light intensity, a 16/8 h photoperiod, and a temperature of 20–25 °C to evaluate its suitability for continued sporophyte development and multiplication. At week 10, gametophytes from all treatments were transferred to the control medium and cultured for an additional 4 weeks. Since the number of initial sporophytes varied among treatments at the time of transfer, sporophyte multiplication efficiency was assessed based on the proportion of sporophytes at transfer (%), proportion of newly induced sporophytes (%), multiplication factor, and the average number of sporophytes at the end of stage 2. The experiment at this stage was carried out in 3 replications. Method for calculation is summarized in Table 1.

**Table 1:** The parameters used for evaluation of development and sporophyte induction in *Phegopteris connectilis* (Michx.) Watt

Parameter	Method of calculation
Stage-1: In vitro gametophyte culture to induce sporophyte	
• Sporophyte Induction Rate (sporophyte/week)	Defined as the increase in the number of sporophytes per unit time (week).
• Specific sporophyte induction rate (sporophytes mg^-1^ of gametophyte week^-1^)	Defined as the rate of formation of new sporophytes per unit of gametophyte biomass per unit time (week), representing the biomass-normalized capacity of gametophytes to initiate sporophyte development.
Stage 2: Subsequent development and increase in the number of sporophytes on gametophytes following in vitro transfer to fresh culture media	
• Multiplication factor	Multiplication factor is defined as the ratio of the number of sporophytes after a given period (or treatment) to the number of sporophytes present at the initial time point, representing the fold change in sporophyte population size.
• Proportion of sporophytes at the time of transfer (%)	Defined as the percentage of sporophytes present at the time of transfer relative to the total number of sporophytes observed after a defined period
• Proportion of newly formed sporophytes (%)	Defined as the percentage of sporophytes formed after transfer relative to the total number of sporophytes observed at the end of the experimental period.
• Average number of sporophytes at the end of stage 2	Average number of sporophytes present at the end of stage 2

The visualisation of effect of nutrient and vitamin concentration on gametophytes and sporophyte induction was made using stereo microscope (ZEISS, Germany) with computer program (ZIESS Labscope) and photographed by digital camera (Nikon D80 equipped with AF-S NIKKOR 18–200 mm f/3.5–5.6G ED VR lens, Nikon Corporation, Tokyo, Japan). Development of filamentous gametophytes was studied by growing gametophyte in liquid MS + V medium. The gametophytes were observed at the interval of 2 weeks under phase-contrast microscope (Eclipse E400, Nikon Corporation, Tokyo, Japan).

### Statistical analysis

Statistical analysis was performed with Statistica software (version 14). One-way analysis of variance (ANOVA) with Tukey’s HSD post hoc test (*p* < 0.05) was performed to compare the results. Pearson correlation analysis was performed to evaluate the relationships among sporophyte production, biomass accumulation, chlorophyll *a* and chlorophyll *b* content, and mineral nutrient content in gametophytes. The analysis was conducted using mean values from the 12 culture treatments as individual observations (*n* = 12). Pearson correlation coefficients (*r*) and corresponding significance levels were calculated, with *p* < 0.05 considered statistically significant. No correction for multiple comparisons was applied, as the correlation analysis was performed as an exploratory assessment of associations among measured physiological parameters.

## Results

### Effect of culture media and vitamins on gametophyte proliferation (stage-1)

**Fig. 2:**
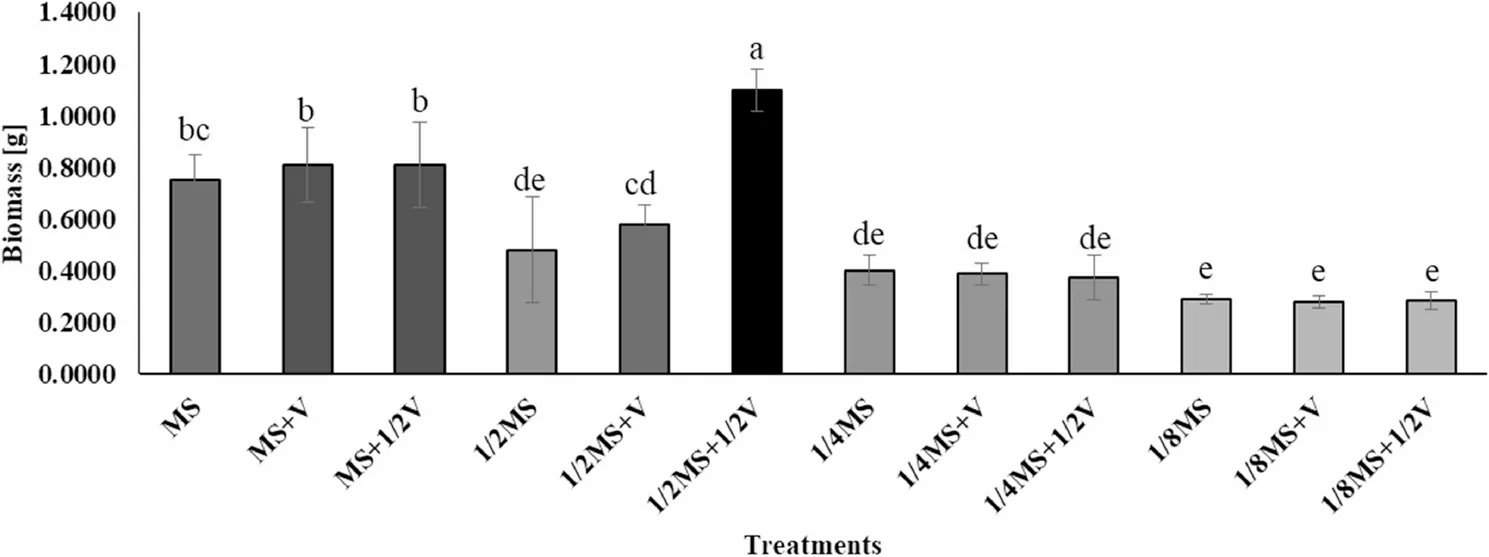
Graph illustrating biomass (g) of *Phegopteris connectilis* gametophytes under different MS medium and vitamin concentrations. The data show the mean of 5 replicates. Treatments followed by the same letter do not differ significantly according to Tukey’s (*p* = 0.05) of ANOVA. Error bars represent the standard deviation (SD) of five biological replicates (*n* = 5). MS – Murashige and Skoog medium; V – vitamins

**Fig. 3:**
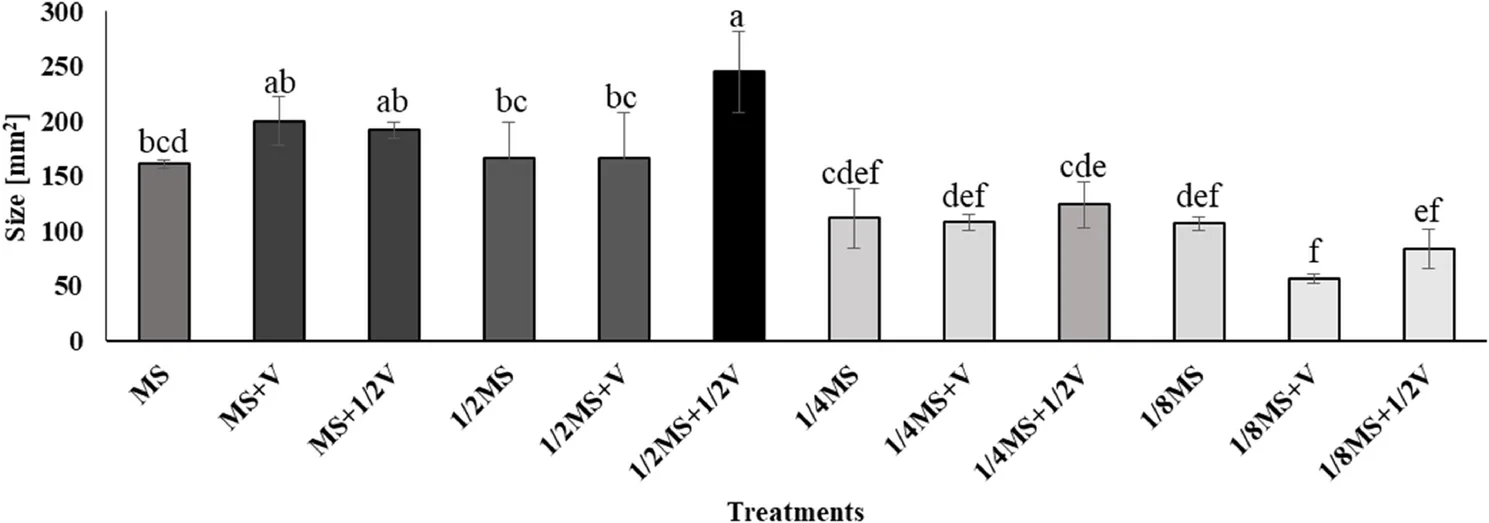
Graph illustrating size (mm^2^) of *Phegopteris connectilis* gametophytes under different MS medium and vitamin concentrations. The data show the mean of 4 replicates. Treatments followed by the same letter did not differ significantly according to Tukey’s (*p* = 0.05) of ANOVA. Error bars represent the standard deviation (SD) of four biological replicates (*n* = 4). MS – Murashige and Skoog medium; V – vitamins

After 10 weeks, gametophytes grown on 1/2MS + 1/2V exhibited significantly higher fresh biomass (1.09 g) than those grown under other treatments (Figs. 1 and 2). This was followed by MS + 1/2V and MS + V with fresh biomasses 0.809 g and 0.808 g (Fig. 2). The pronounced effect of vitamin was evident only in full and 1/2 MS medium concentration. Substantial decrease in biomass was observed with 1/4MS and 1/8MS medium irrespective of vitamin concentration (Fig. 2). In the same line, increase in the size (245 mm^2^) was reported with 1/2MS + 1/2V (Fig. 3). Although decrease in the size of gametophyte cluster was observed in 1/4MS and 1/8MS, with the presence of vitamin the gametophytes exhibited larger cluster size in all media concentrations, apart from 1/8 MS (Fig. 3). In addition, gametophytes cultured on 1/4 MS and 1/8 MS media showed signs of necrosis, whereas green gametophytes bearing sporophytes and without visible signs of necrosis were observed on MS and 1/2 MS media (Fig. 1).

### Effect of culture media and vitamins on induction of sporophyte (stage-1)

**Fig. 4:**
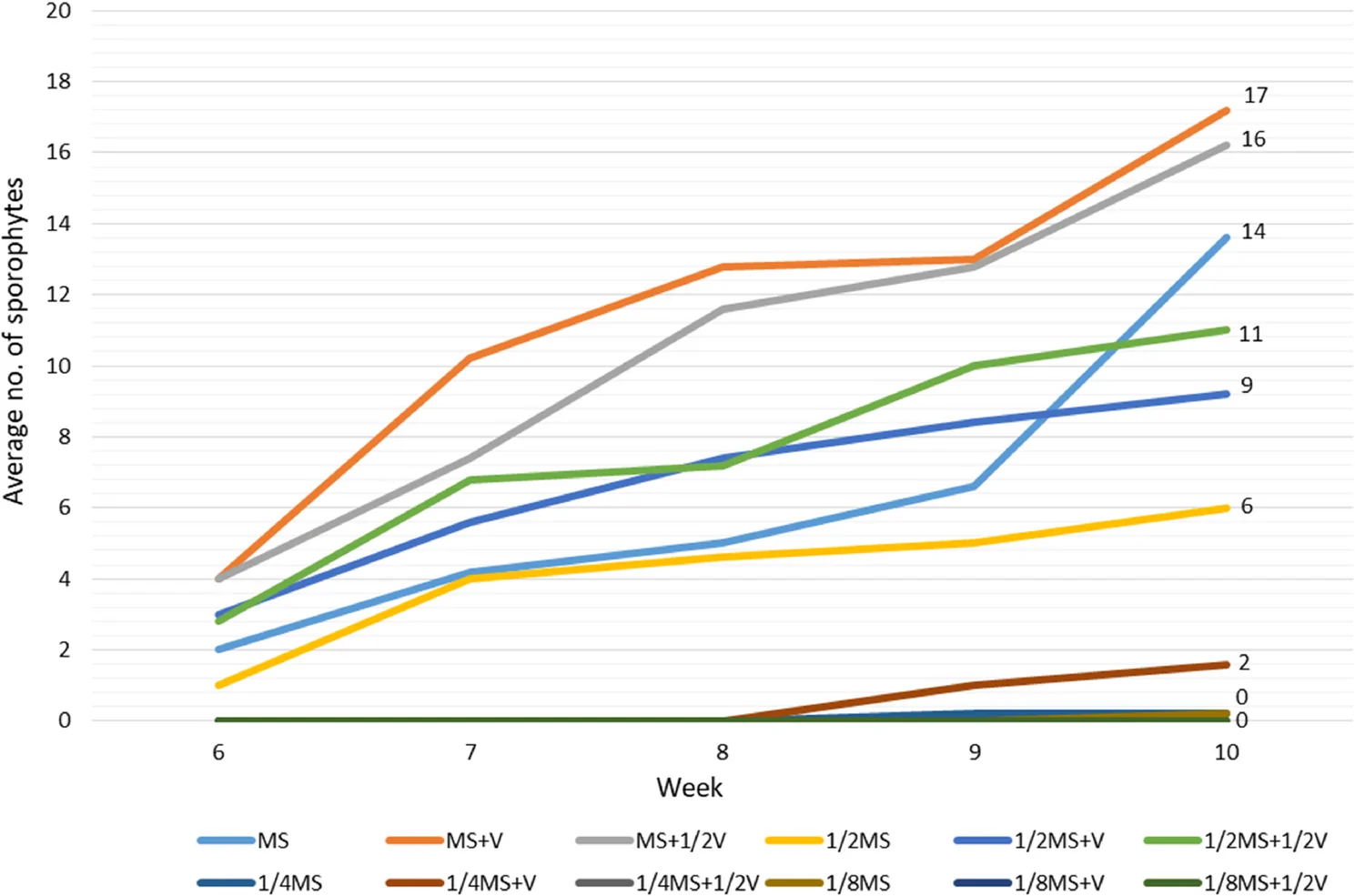
Graph depicting average number of sporophytes in stage-1 (5 replications) produced by *Phegopteris connectilis* gametophytes under different MS medium and vitamin concentrations. One-way ANOVA followed by Tukey’s HSD post hoc test indicated significant differences among treatments (*p* < 0.05). The corresponding mean ± standard deviation values and statistical analysis are provided in Supplementary Table S1. MS – Murashige and Skoog medium; V – vitamins

The first sporophytes were observed during the 6th week of gametophyte culture in the MS, MS + V, MS + 1/2V, 1/2MS, 1/2MS + V, and 1/2MS + 1/2V treatment combinations (Fig. 4). During the 6th week, an increase in the number of sporophytes (4 per plate) was recorded in gametophytes grown in MS + V and 1/2MS + 1/2V. However, the number of sporophytes did not differ significantly with the gametophytes grown on MS and 1/2MS, with or without vitamins. After 10 weeks, the highest number of sporophytes was observed in MS + V, followed by MS + 1/2V and MS with the average number of sporophytes being equal 17, 16, and 14 per petriplate, respectively. However, there was no significant difference in the number of sporophytes between gametophytes grown on MS and 1/2MS, with or without vitamins. The highest specific sporophyte induction rate was observed in MS + V (0.068 sporophytes mg^− 1^ of gametophyte week^− 1^). These results indicated that, gametophyte proliferation was maximized in 1/2MS + 1/2V, whereas full strength MS medium supplemented with vitamins (MS + V) showed a trend toward higher sporophyte production under tested conditions in *Phegopteris connectilis.*

### Effect of culture media and vitamins on chlorophyll and carotenoid content in gametophytes (stage-1)

**Fig. 5:**
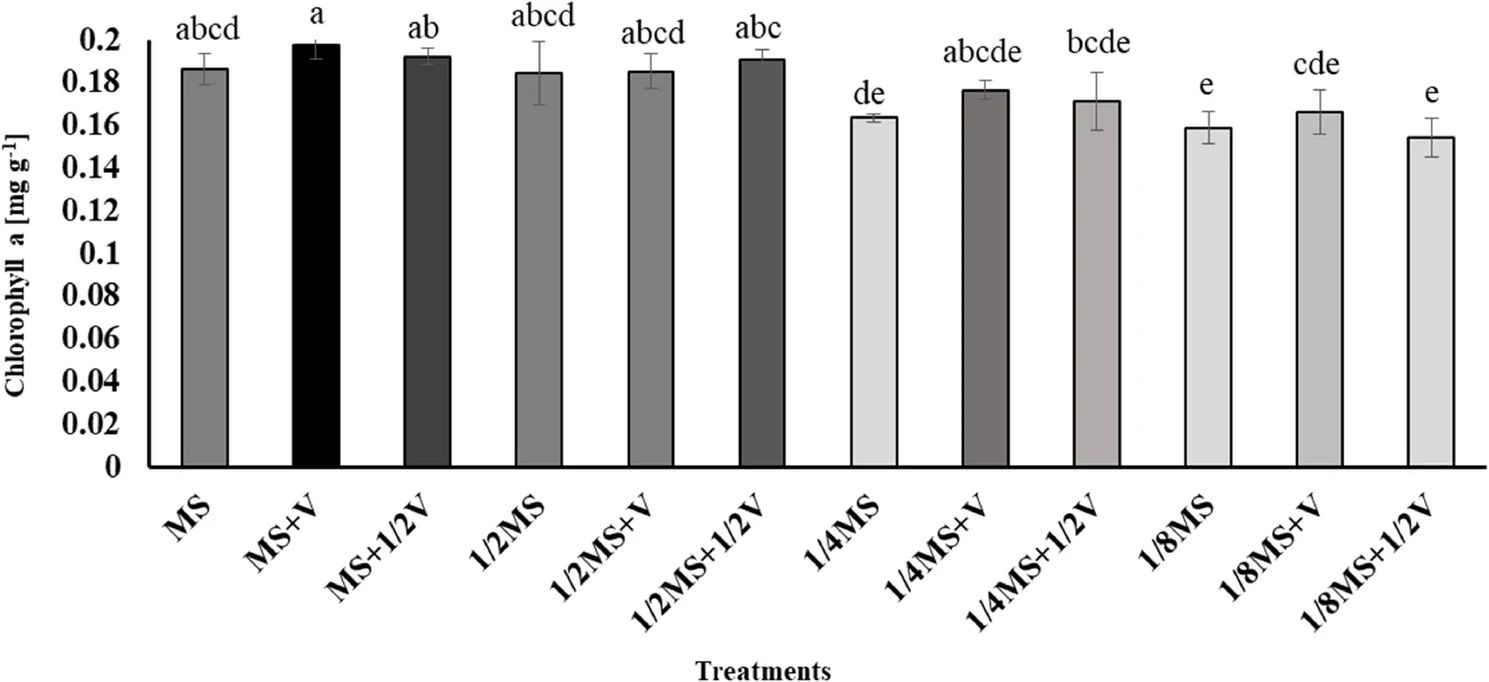
Graph presenting chlorophyll a content (mg g^− 1^) in *Phegopteris connectilis* gametophytes under different MS medium and vitamin concentrations. The data show the mean of 3 replicates, treatment followed by the same letter did not differ significantly according to Tukey’s (*p* = 0.05) of ANOVA. Error bars represent the standard deviation (SD) of three biological replicates (*n* = 3). MS – Murashige and Skoog medium; V – vitamins

**Fig. 6:**
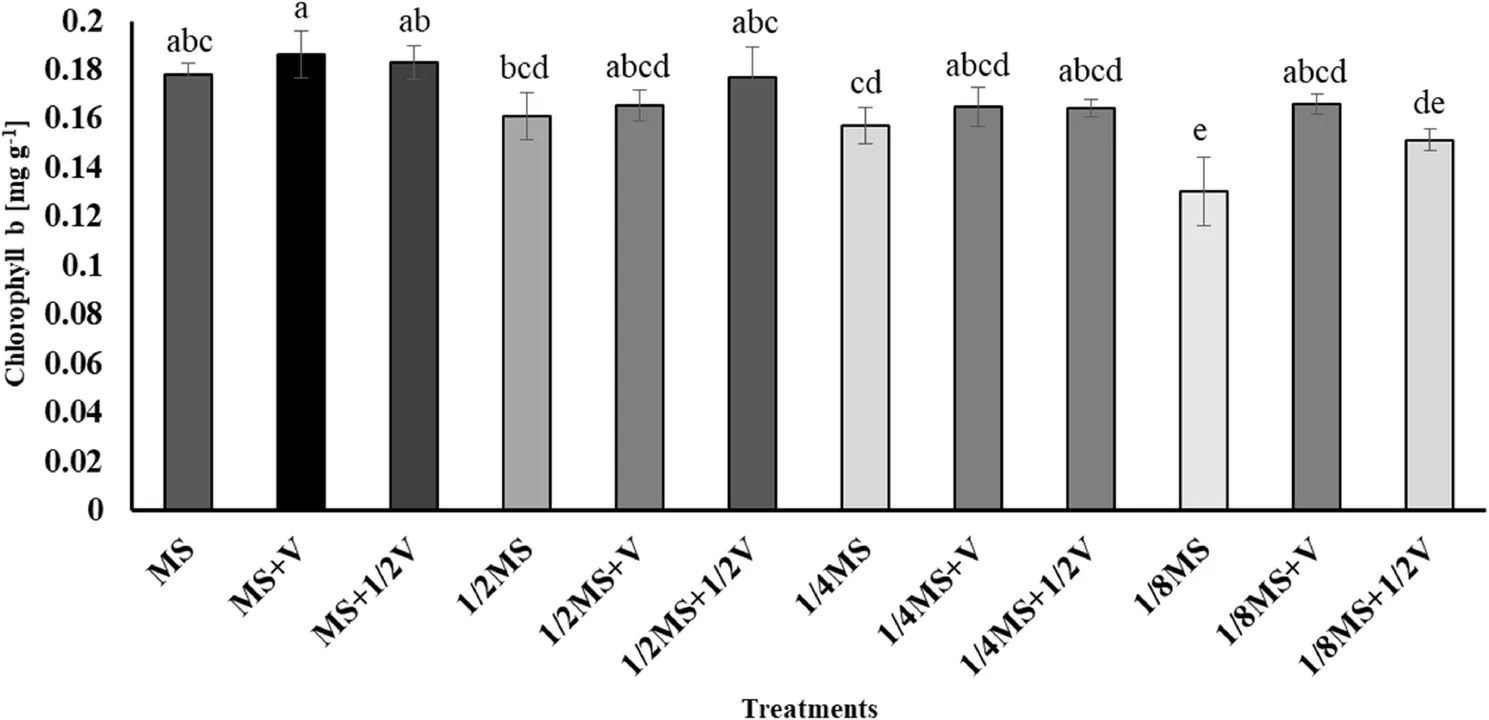
Graph presenting chlorophyll b content (mg g^− 1^) in *Phegopteris connectilis* gametophytes under different MS medium and vitamin concentrations. The data show the mean of 3 replicates, treatment followed by the same letter did not differ significantly according to Tukey’s (*p* = 0.05) of ANOVA. Error bars represent the standard deviation (SD) of three biological replicates (*n* = 3). MS – Murashige and Skoog medium; V – vitamins

**Fig. 7:**
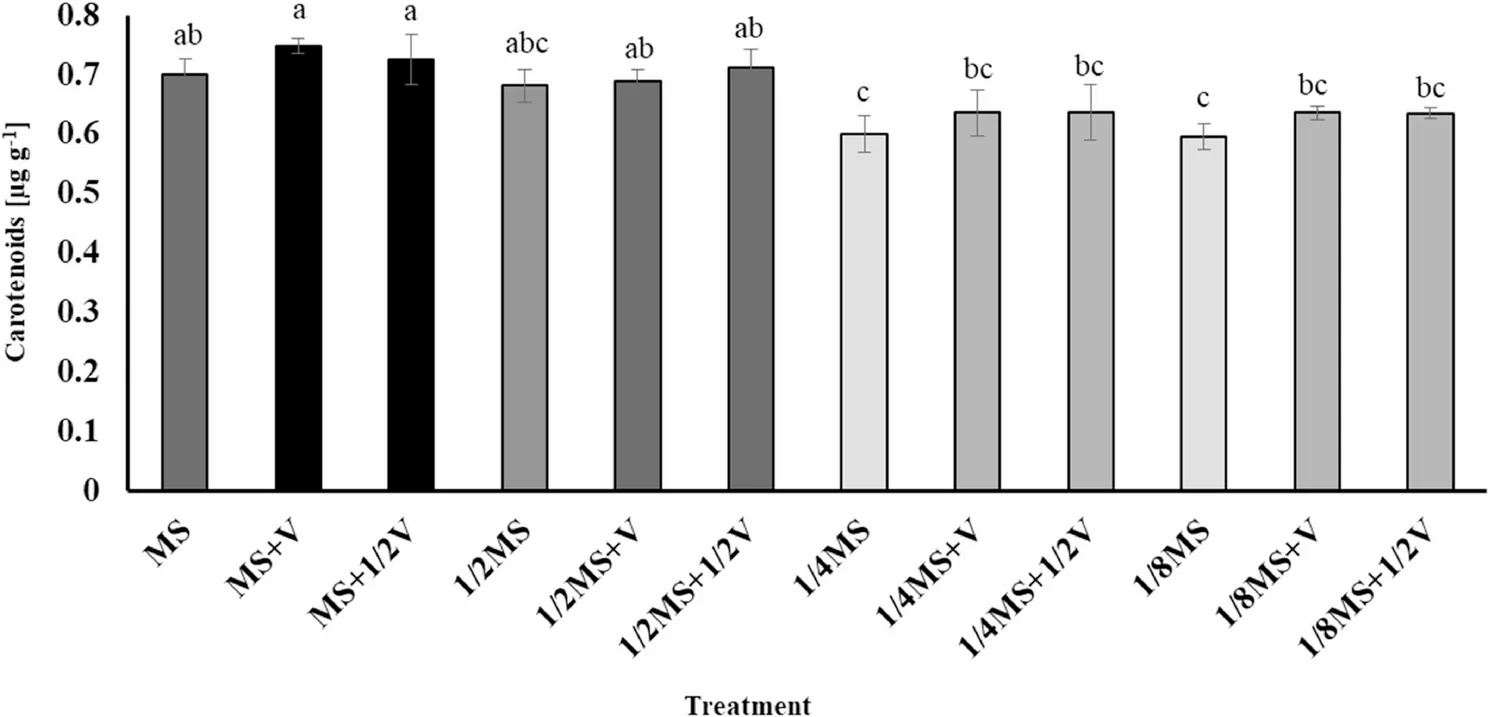
Graph exhibiting carotenoid content (µg g^− 1^) in *Phegopteris connectilis* gametophytes under different media and vitamin concentrations. The data show the mean of 3 replicates, treatment followed by the same letter did not differ significantly according to Tukey’s (*p* = 0.05) of ANOVA. Error bars represent the standard deviation (SD) of three biological replicates (*n* = 3). MS – Murashige and Skoog medium; V – vitamins

An increase in chlorophyll a and b (mg g^− 1^ fresh weight) was observed in gametophytes cultured on MS + V medium as compared to other treatments under study (Figs. 5 and 6). An increase in carotenoid content (µg/g fresh weight) was observed in the MS + V treatment; however, it did not differ significantly from other full-strength MS treatments (MS and MS + 1/2V) or half-strength MS treatments (1/2MS, 1/2MS + V, and 1/2MS + 1/2V) (Fig. 7). Among other treatments, with decrease in the strength of MS medium, there was a decrease in chlorophyll and carotenoid content in fern gametophytes. The positive effect of vitamin supplementation on chlorophyll and carotenoid content in developing gametophytes was evident as application of vitamins diminished concentration-dependent decline in pigment content across all MS medium strengths tested.

### Effect of culture media and vitamins on nutrient content of gametophytes (stage-1)

On analyzing the nutrient content in gametophytes after 10 weeks, MS + V showed a notable increase in concentration of macronutrients namely, Ca, K & Mg and micronutrients namely, Zn, Fe & Mn (Tables 2 and 3). However, a significant increase was reported only in K, Mg, &Mn only in this treatment as compared to other treatments under study.

**Table 2:** Macro-nutrient content (mg/100 g of dry weight) of *Phegopteris connectilis* gametophytes under different MS medium and vitamin concentrations

Macro-nutrient content in gametophytes (mg/100 g dried biomass)						
Treatment	**N**	**P**	**K**	**Ca**	**Mg**	**Na**
MS	3722^a^±98	163^a^±20.44	2394^b^±131.61	404^b^±25.33	133^b^±7.28	173^cd^±11.76
MS + V	2978^b^±122	153^a^±14.35	2562^a^±60.19	477^a^±16.29	145^a^±2.28	183^bc^±4.47
MS + 1/2V	2665^bc^±113	155^a^±2.49	2254^bc^±72.48	452^a^±14.52	131^b^±5.61	169^de^±1.65
1/2MS	2391^cd^±42	102^bc^±19.36	1199^e^±23.83	326^d^±6.91	88^e^±2.46	149^gh^±2.45
1/2MS + V	2208^cd^±132	93^c^±9.84	1449^d^±21.7	362^c^±5.52	97^d^±1.21	188^b^±1.96
1/2MS + 1/2V	2405^cd^±145	134^ab^±6.09	2114^c^±52.16	469^a^±14.45	115^c^±2.15	204^a^±6.03
1/4MS	2171^de^±51	86^c^±12.24	759^fg^±8.21	229^fg^±2.61	71^hi^±1.37	151^fgh^±0.75
1/4MS + V	2013^def^±175	88^c^±8.28	902^f^±7.17	253^ef^±1.66	78^fgh^±0.45	154^fg^±1.26
1/4MS + 1/2V	1553^fg^±33	76^c^±9.35	782^fg^±14.53	238^f^±3.39	69^i^±1.17	152^fgh^±2.89
1/8MS	1215^gh^±186	77^c^±4.41	663^g^±2.31	198^g^±0.41	75^ghi^±0.97	141^h^±1.89
1/8MS + V	927^h^±334	98^c^±11.07	886^f^±22.26	241^f^±4.8	85^ef^±1.28	163^def^±3.58
1/8MS + 1/2V	1700^ef^±245	89^c^±5.71	1087^e^±19.9	275^e^±3.81	82^efg^±0.66	158^efg^±1.44

Values are presented as mean ± standard deviation (SD) of biological replicates. The data show the mean of 3 replicates. Treatments followed by the same letter did not differ significantly according to Tukey’s (*p* = 0.05) of ANOVA. MS – Murashige and Skoog medium; V – vitamins

**Table 3:** Micro-nutrient content (mg/100 g of dry weight) of *Phegopteris connectilis* gametophytes under different MS medium and vitamin concentrations

Micro-nutrient content in gametophytes (mg/100 g dried biomass)					
**Treatment**	**Zn**	**Fe**	**Mn**	**Cu**	**B**
MS	7.96^a^±0.47	27^ab^±1.55	10.1^d^ ± 0.47	0.75^abc^±0.12	9.08^a^±1.13
MS + V	8.33^a^±0.17	28^a^±0.51	12.1^a^ ± 0.06	0.833^ab^±0.17	7.23^b^±0.17
MS + 1/2V	8.2^a^ ± 0.6	26^b^±0.55	10.6^c^ ± 0.15	1.033^a^±0.15	6.57^bc^±0.35
1/2MS	5.54^cd^±0.17	19^d^±0.57	7.7^f^ ± 0.06	0.465^bc^±0.3	4.69^de^±0.37
1/2MS + V	5.8^c^±0	16^e^±0.15	8.3^e^ ± 0.01	0.659^abc^±0.06	5.13^cd^±0.11
1/2MS + 1/2V	6.93^b^±0.35	23^c^±0.65	11.5^b^ ± 0.32	0.833^ab^±0.14	6.83^b^±0.25
1/4MS	3.47^f^±0.2	11^hi^±0.29	4.7^hi^±0.15	0.62^bc^±0.06	3.87^de^±0.83
1/4MS + V	3.9^ef^±0.36	13^fg^±0.2	5^h^±0.01	0.567^bc^±0.05	3.57^de^±0.15
1/4MS + 1/2V	3.58^f^±0.06	12^gh^±0.37	5.8^g^ ± 0.06	0.5^bc^±0.1	3.89^de^±0.26
1/8MS	3.76^ef^±0.26	10^i^±0.17	3.3^j^ ± 0.01	0.5^bc^±0.1	3.18^e^±0.24
1/8MS + V	4.61^de^±0.17	12^gh^±0.64	4.4^i^ ± 0.11	0.4^c^ ± 0.1	3.53^de^±0.17
1/8MS + 1/2V	4.33^ef^±0.38	14^ef^±0.45	5.5^g^ ± 0.07	0.347^c^±0.06	4.38^de^±1.08

Values are presented as mean ± standard deviation (SD) of biological replicates. The data show the mean of 3 replicates. Treatments followed by the same letter did not differ significantly according to Tukey’s (*p* = 0.05) of ANOVA. MS – Murashige and Skoog medium; V – vitamins

When considering full strength of MS (MS) medium, presence of vitamins significantly affected N content, wherein N and B content was significantly higher in MS alone. While the presence of vitamins did not significantly vary P and Zn content. Cu on the contrary, was highest in MS with half dose of vitamins. Ca content did not vary significantly between MS + V, MS + 1/2V and 1/2MS + 1/2V, however, mere presence of vitamins depicted a significant increase when compared to MS alone.

On half strength of MS (1/2MS) medium supplemented with half dose of vitamins, gametophytes demonstrated a significantly higher Na concentration as compared to other treatments under study. Herein, vitamin supplementation did not significantly vary N content. Moreover, gametophytes cultured on 1/2MS + 1/2V treatment showed a significant increase in K, Mg, Zn, Fe, Mn, and B as compared to 1/2MS + V and 1/2MS.

Taking 1/4MS into account, full dose of vitamins enhanced K, Ca, Mg, Na, Zn and Fe content as compared to 1/4MS alone and 1/4MS with half dose of vitamins. However, N content was higher in 1/4MS alone.

Similarly, in case of 1/8MS, N content was reported to be higher in 1/8MS with half dose of vitamin supplementation. Full dose of vitamins positively influenced Mg, Na and Zn content, whereas, half dose of vitamins resulted in higher K, Ca, Fe, Mn and B content.

**Fig. 8:**
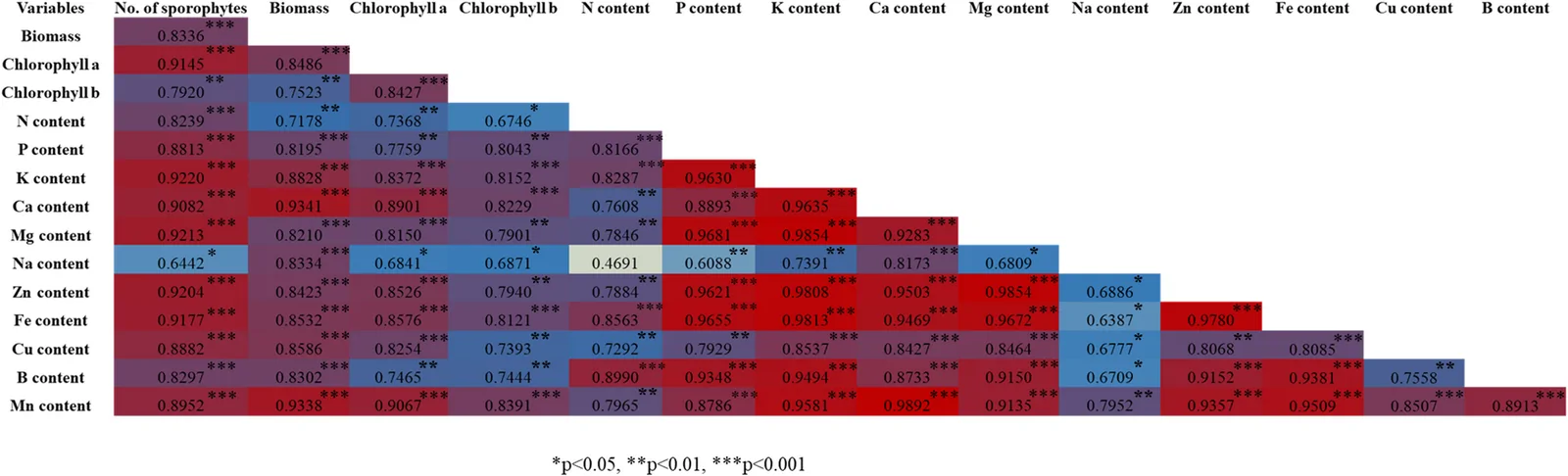
Heat map of Pearson correlation coefficients (*r*) of plant physical and physiological traits. Color scale indicates both the magnitude and direction of correlations: cream denotes weak correlations, blue indicates moderate correlations, and red represents strong positive correlations (indicated by the scale on left). *p<0.05, **p<0.01, ***p<0.001

All analysed nutrients, except Na (*r* = 0.64), showed very strong positive correlations with sporophyte production (*r* = 0.82–0.92), and all relationships were highly significant (*p* < 0.001). This indicates that increased nutrient content was correlated with enhanced sporophyte production (Fig. 4 and 8). Although the positive correlation suggests a potential association between nutrient availability and sporophyte production, further experimental studies are required to determine whether nutrient availability directly influences sporophyte induction. In case of biomass growth the correlation was slightly weaker. Only for Ca and Mn *r* achieved value 0.93, for the remaining nutrients correlation amounted from 0.72 (for N) to 0.88 (for K).

### Subsequent development and increase in the number of sporophytes on gametophytes following in vitro transfer to fresh culture media (stage-2)

**Fig. 9:**
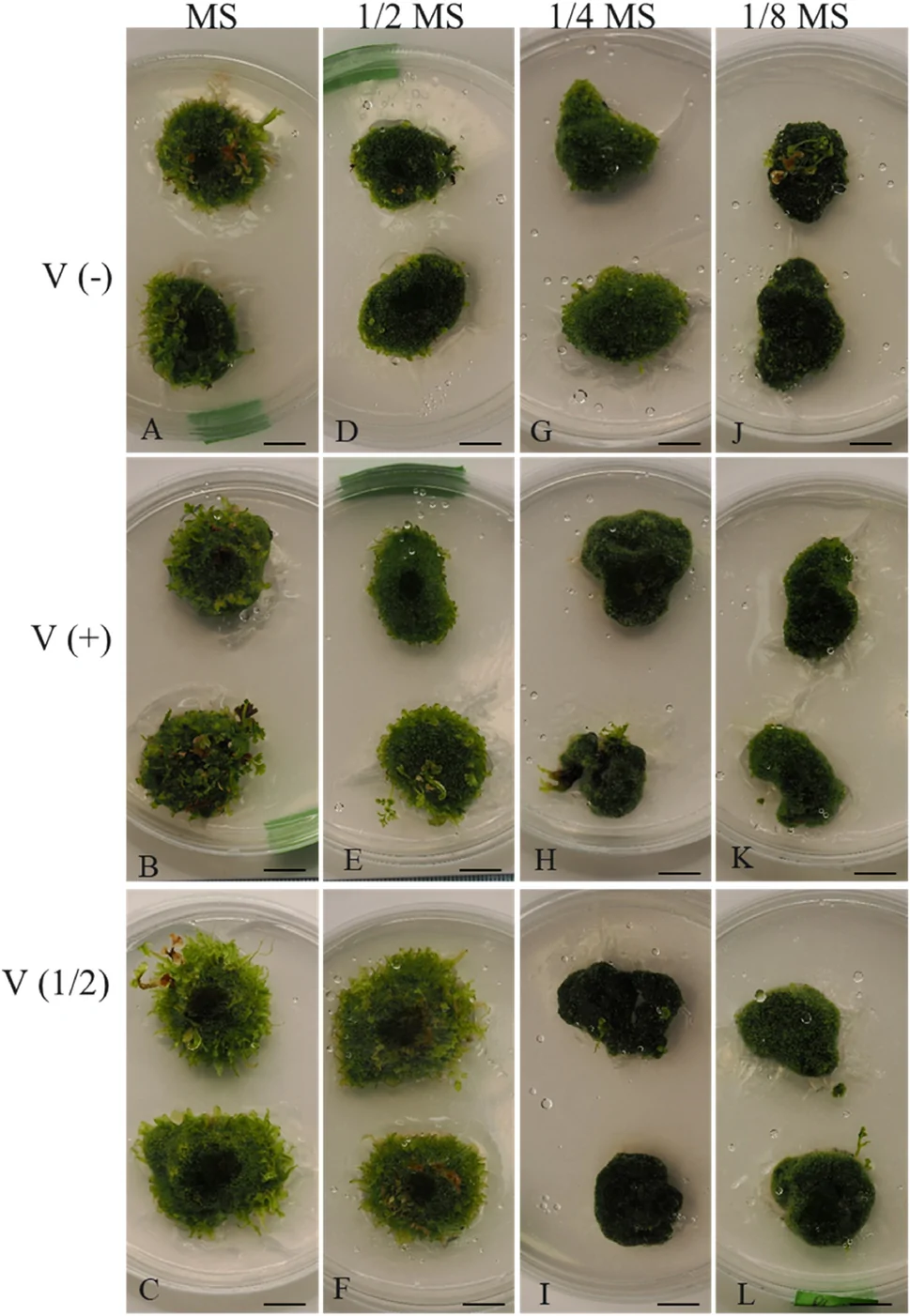
Effect of different MS medium and vitamin concentrations on subsequent development and increase in the number of sporophytes on gametophytes following in vitro transfer to fresh culture media (**A**–**L**) Scale bar = 1 cm. MS – Murashige and Skoog medium; V – vitamins

**Fig. 10:**
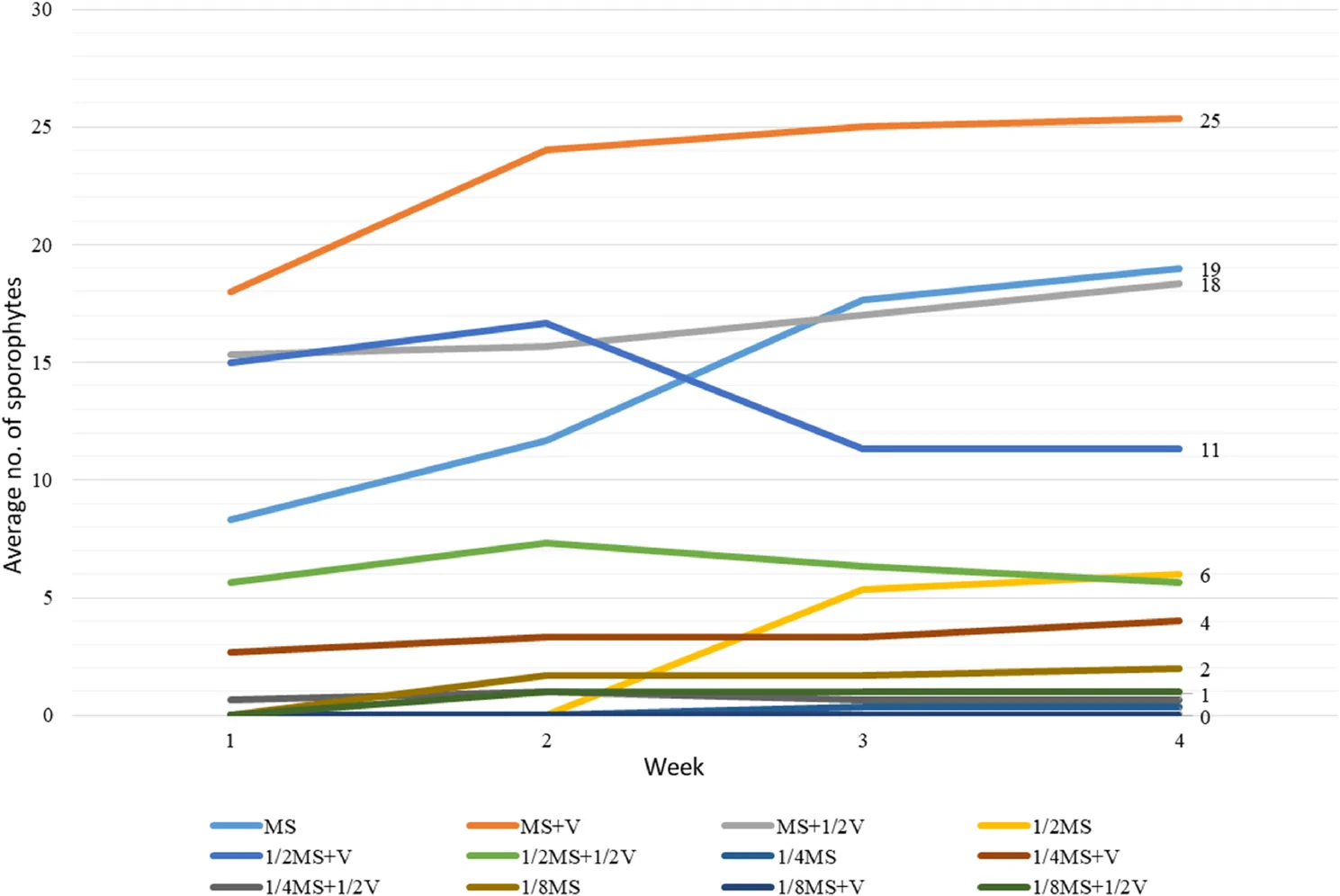
Graph depicting average number of sporophytes developed in Stage-2 (3 replications) produced by *Phegopteris connectilis* gametophytes. One-way ANOVA followed by Tukey’s HSD post hoc test indicated significant differences among treatments (*p* < 0.05). The corresponding mean ± standard deviation values and statistical analysis are provided in Supplementary Table S2. MS – Murashige and Skoog medium; V – vitamins

All sporophytes with gametophytes survived transfer to reference control medium. Moreover, treatments that exhibited signs of necrosis due to nutrient limitation recovered from the stressed condition, as indicated by the reappearance of green tissue (Figs. 9). Nevertheless, the sporophytes grown initially on MS alone when transferred to reference control medium demonstrated 2.29-fold post-transfer sporophyte formation (multiplication factor), which was highest among the treatments (Table 5). However it did not differ significantly from the post-transfer sporophyte multiplication observed in gametophytes cultured on MS and 1/2MS with or without vitamin supplementation. Only 1.41 fold post-transfer sporophyte multiplication was registered with MS + V treatment (Table 5). However, highest average number of sporophytes was obtained in MS + V at the end of stage 2 (Fig. 10, Supplementary table S2).

The results suggest that the MS + V medium promoted early initiation of sporophytes, with a specific induction rate of 0.068 sporophytes mg⁻¹ week⁻¹ during stage 1 (Table 4). This early induction was followed by a relative decline in the formation of new sporophytes during stage 2 compared with the MS treatment.

In contrast, cultures grown in MS medium exhibited a slightly lower initial induction rate (0.058 sporophytes mg⁻¹ week⁻¹) during stage 1, but sporophyte formation enhanced after transfer to the reference control medium in stage 2 (Tables 4 and 5). This pattern suggested a compensatory growth response, where gametophytes initially grown in MS medium increase sporophyte production after being transferred to medium with vitamins. In addition, gametophytes cultured on 1/4 MS and 1/8 MS media with and without vitamins exhibited a recovery response characterized by the reappearance of green tissue and reduction of visible necrosis, which may represent a compensatory growth response. Moreover, gametophytes from the treatments 1/4MS + V, 1/4MS + 1/2V, 1/8MS and 1/8MS + 1/2V also demonstrated sporophyte production at the end of stage-2.

**Table 4:** Specific sporophyte induction rate at the end of stage-1

Treatment	Induction Rate (sporophyte/week)	Specific sporophyte induction rate (sporophytes mg^− 1^ gametophyte week^− 1^)
MS	2.9^a^ ± 2.44	0.058^a^±0.04
MS + V	3.4^a^ ± 3.27	0.068^a^±0.06
MS + 1/2V	3.05^a^±1.78	0.061^a^±0.03
1/2MS	1.15^ab^±2.98	0.025^ab^±0.04
1/2MS + V	1.45^ab^±1.55	0.029^ab^±0.03
1/2MS + 1/2V	2.05^ab^±3.09	0.041^ab^±0.06
1/4MS	0.05^b^±0.11	0.001^b^±0
1/4MS + V	0.4^b^ ± 0.89	0.008^b^±0.01
1/4MS + 1/2V	0^b^±0	0^b^±0
1/8MS	0.05^b^±0.11	0.001^b^±0
1/8MS + V	0^b^±0	0^b^±0
1/8MS + 1/2V	0^b^±0	0^b^±0

Values are presented as mean ± standard deviation (SD) of biological replicates. The data show the mean of 5 replicates. Treatments followed by the same letter did not differ significantly according to Tukey’s (*p* = 0.05) of ANOVA. *MS* Murashige and Skoog medium, *V *Vitamins

**Table 5:** Sporophyte formation and increase on gametophyte after in vitro gametophyte transfer at the end of stage 2

Treatment	Multiplication factor	Proportion of sporophytes at the time of transfer (%)-developed in stage-I	Average number of sporophytes at the end of stage 2	Proportion of newly formed sporophytes (%)-developed in stage-II
MS	2.29^a^±0.35	44^bc^±7.27	19^ab^±4.04	56
MS + V	1.41^ab^±0.17	71^abc^±9.56	25^a^±4.93	28
MS + 1/2V	1.19^abc^±0.05	83^ab^±3.86	18^ab^±2.3	16
1/2MS	0^c^±0	0^c^±0	6^bc^±10.39	33
1/2MS + V	0.75^bc^±0.18	140^a^±39.76	11^bc^±6.08	-40
1/2MS + 1/2V	1.0^abc^±0.22	95^ab^±18.02	6^bc^±3.78	5
1/4MS	0^c^±0	0^c^±0	0.3^c^ ± 0.57	33
1/4MS + V	1.14^abc^±1.03	60^bc^±52.91	4^c^±5.29	14
1/4MS + 1/2V	0.67^bc^±1.15	50^bc^±50	1^c^±1	17
1/8MS	0^c^±0	0^c^±0	2^c^±3.46	33
1/8MS + V	0^c^±0	0^c^±0	0^c^±0	0
1/8MS + 1/2V	0^c^±0	0^c^±0	1^c^±1.73	33

Values are presented as mean ± standard deviation (SD) of biological replicates. The data show the mean of 3 replicates. Treatments followed by the same letter did not differ significantly according to Tukey’s (*p* = 0.05) of ANOVA. The proportion of newly formed sporophytes developed in stage II did not differ significantly among treatments. *MS* Murashige and Skoog medium, *V* Vitamins

### Morphology of gametophyte in response to nutrient concentration and sporophyte induction

**Fig. 11:**
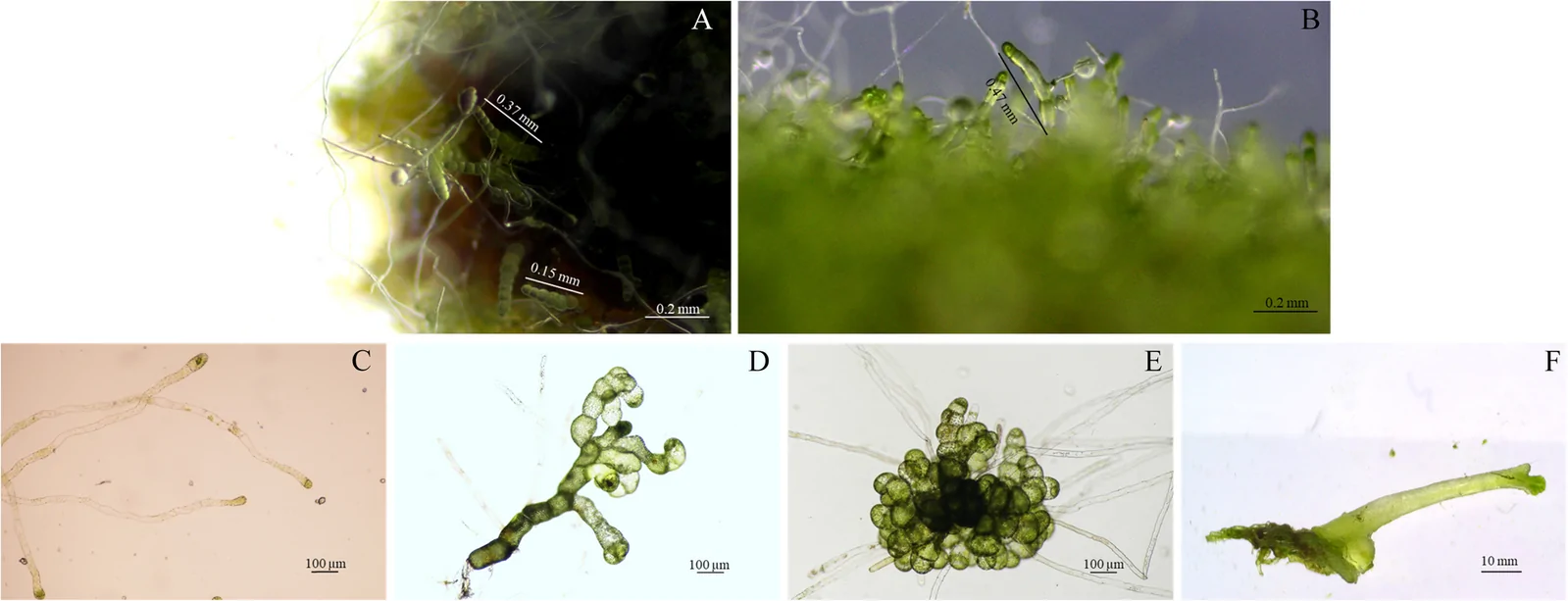
Growth patterns and developmental progression from filamentous gametophyte to sporophyte in *Phegopteris connectilis*. **A** Representative image showing filament length under MS and 1/2 MS conditions, (**B**) Representative image showing filament length under 1/4 MS and 1/8 MS conditions, (**C**) Filamentous gametophyte stage, (**D**) Transverse cell division within the filament forming branches, (**E**) Cluster of filaments undergoing active proliferation under favorable conditions, (**F**) Developmental progression from gametophyte growth to sporophyte emergence

**Fig. 12:**
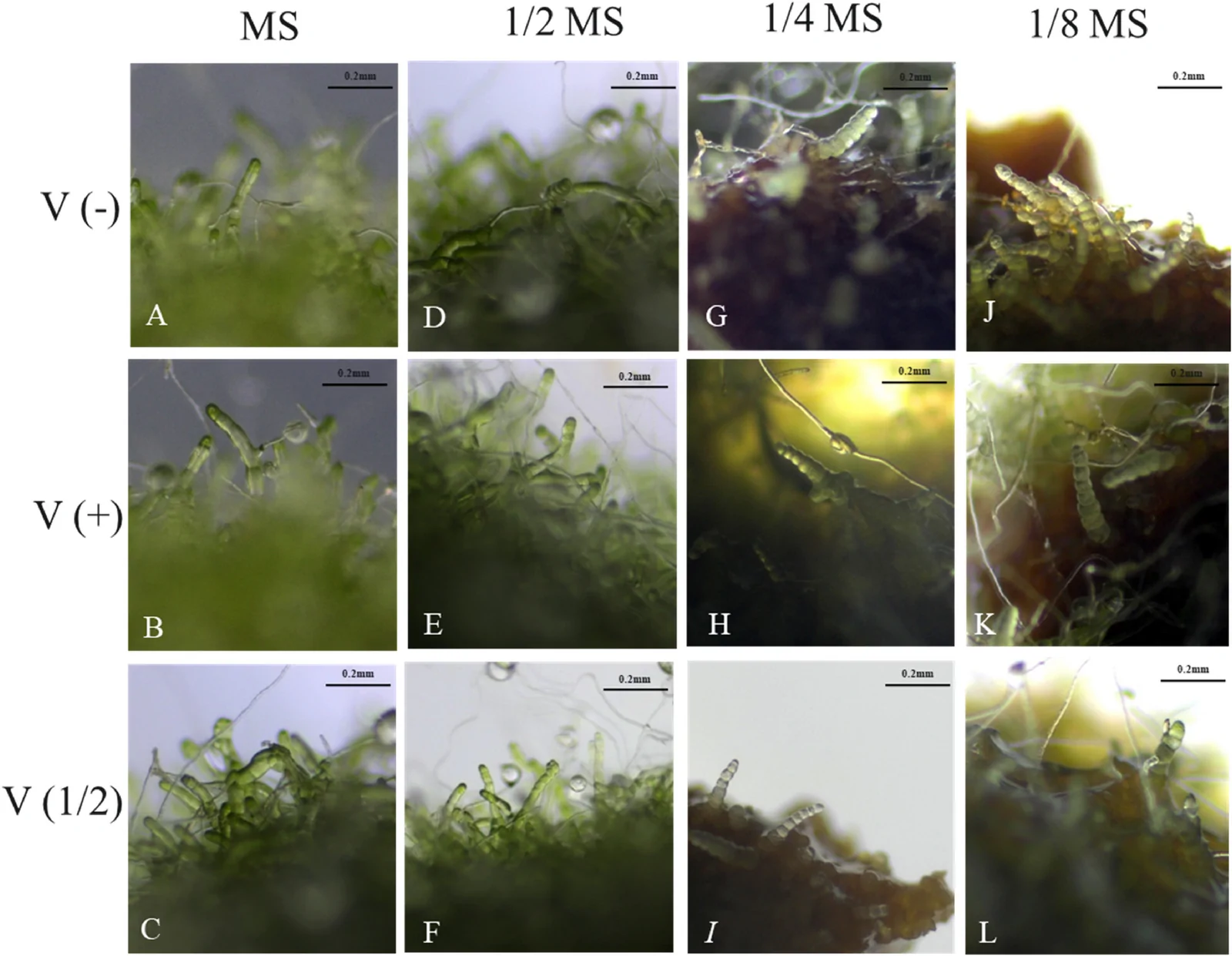
Qualitative differences in filamentous gametophyte morphology under different mineral nutrient and vitamin concentration. **A**-**F** Gametophytes cultured on MS and 1/2 MS with relatively elongated/cylindrical cells in filament. Gametophytes cultured on 1/4 MS and 1/8 MS with more compact arrangement of cells in filament. MS – Murashige and Skoog medium; V – vitamins

**Fig. 13:**
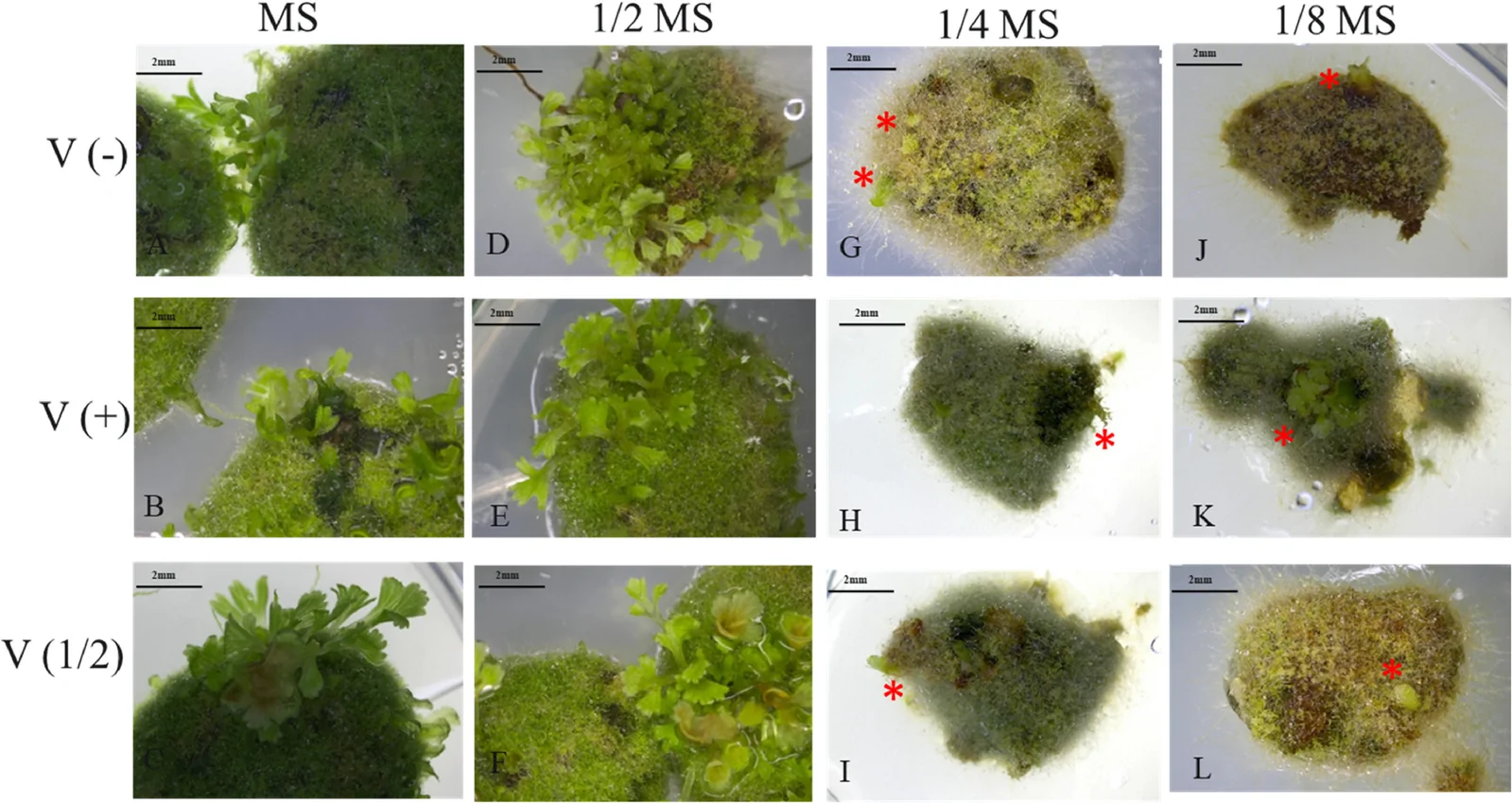
Effect of nutrient and vitamin concentration on development of rhizoids. * indicate thicker and more compact gametophyte clusters developed under nutrient-limited conditions. MS – Murashige and Skoog medium; V – vitamins

*Phegopteris connectlis* gametophytes are branched filaments, forming mats in bright green color. A distinct difference in the overall mat structure and cellular orientation was detected between gametophytes cultured on treatments with varying strength of MS medium irrespective of vitamin supplementation. Each filament consisted of cylindrical cells, with length of filaments ranging from 0.15 to 0.37 mm when grown in nutrient limiting condition (1/4MS and 1/8MS irrespective of vitamin supplementation) and 0.47 mm long when grown under sufficient nutrient supply (MS and 1/2MS irrespective of vitamin supplementation.) (Fig. 11). Altered cell morphology, characterized by visible cellular compaction, was observed in 1/4 MS and 1/8 MS conditions irrespective of the presence or absence of vitamins in the medium (Fig. 12). These qualitative morphological observations indicated reduced cell expansion while cell division appeared to continue. Consequently, cells appeared smaller and more densely packed under 1/4 MS and 1/8 MS conditions. This led to the development of thicker and more compact gametophyte clusters depicted in Fig. 13. Furthermore, nutrient limitation not only influenced cellular compaction, but also led to increase in rhizoid formation. Figure 13 demonstrates increased and elongated rhizoids in 1/4MS and 1/8MS. Rhizoids were unbranched and non-gravitropic (Fig. 13).

**Fig. 14:**
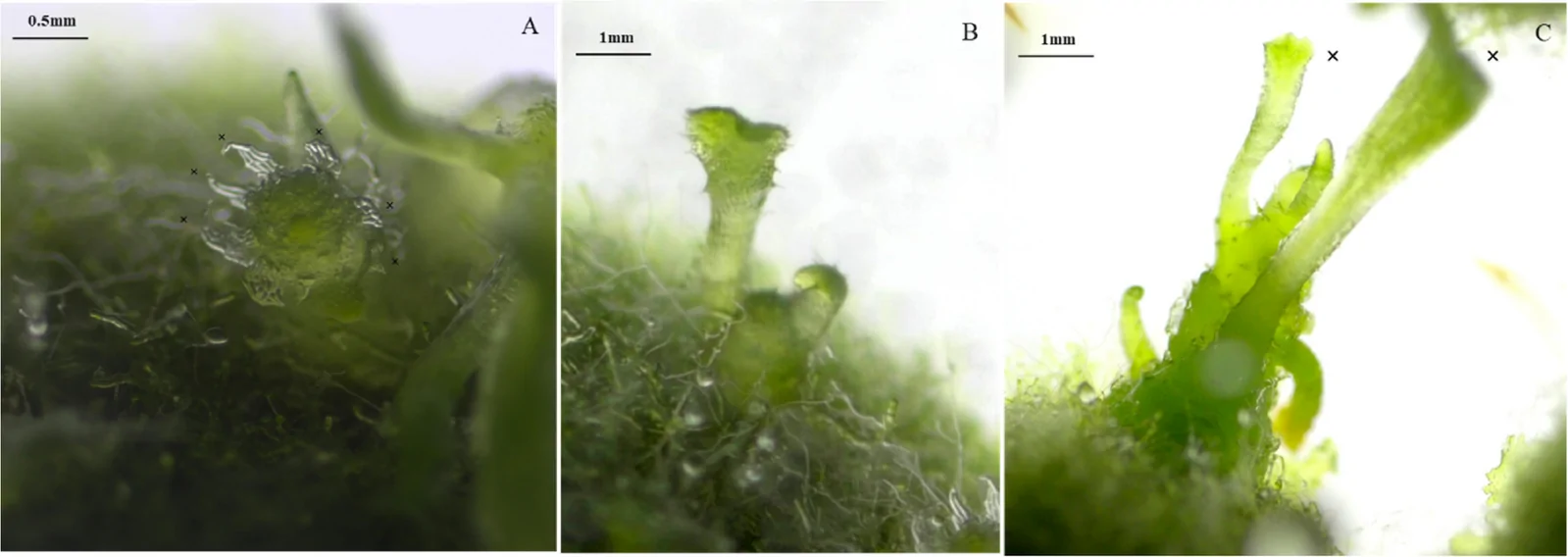
Development of a sporophyte of *Phegopteris connectilis.* **A** The apical view of the spirally arranged leaf primordia around the apex. **B** A juvenile sporophyte leaf and a curled fiddlehead resembling young sporophyte of *Phegopteris connectilis* formed on a bud-like structure. **C** Two sporophyte leaves emerging from a bud-like structure

As mentioned previously, in the 6th week of culture, sporophytes emerged on gametophytes. They emerged from a bud-like structure developed in the mass of gametophyte (Fig. 14). The bud possessed leaf primordia arranged spirally around the apex meristem (Fig. 14A). Gradually, the bud bore leaves of juvenile sporophyte as depicted in Fig. 14B and C.

## Discussion

This study provides insights into in vitro development protocols for *Phegopteris connectilis* from gametophytes. *Phegopteris connectilis* gametophytes appeared as branched filaments, forming mats that were bright green. *Phegopteris connectilis* is an apogamous fern, wherein development of sporophyte occurs without fertilization. This study used different combination of MS and vitamins to identify most effective medium under tested condition for sporophyte induction, formation and growth on gametophyte. First sporophytes emerged after 5 weeks of culture. Herein, highest induction rate of sporophytes was observed in MS + V medium. Previous study on this species reported the formation of apogamous embryo after seven weeks from the initiation of the culture through green sporangia (sori) [24]. In this study the gametophytes were in protofilament stage. A similar appearance of gametophytes has been observed in the species *Trichomanes speciosum* Willd., and *Trichomanes holopterum* Kunze belonging to the family Hymenophyllaceae [33, 34]. Moreover, herein sporophyte emerged from a bud-like structure. These ‘buds’ could correspond to the epiphyllous buds discussed previously in ferns [35, 36]. Since the emergence of epiphyllous buds has not been discussed in *Phegopteris connectilis*, further studies must be carried out to understand the emergence of bud-like structures in this species.

Numerous factors influence apogamic sporophyte induction. Apogamy has been previously associated with sugar content in the medium. Initial studies on apogamy in ferns namely, *Dryopteris affinis* (Lowe) Fraser-Jenk., *Osmunda cinnamomea* L., *Dennstaedtia punctilobula* (Michx.) T. Moore, *Dryopteris marginalis* (L.) A. Gray, etc. have indicated the importance of sucrose in media to induce apogamy as it regulates osmotic potential of medium [22, 37]. The role of ethylene in inducing apogamy in *Pteridium aquilinum* (L.) Kuhn has also been reported [38, 39]. Later Menéndez et al., 2006 revealed the role of auxins and gibberellins in the development of apogamous embryo in *Dryopteris affinis*. Furthermore, pivotal role of either macronutrients namely, N, P, Mg, Ca, Na or micronutrients namely, Mn, Cu, Zn, B, Mo, Fe in governing sporophyte induction and sex gametes expression independent of sucrose has been demonstrated in apogamous fern *Actiniopteris radiata* (Sw.) Link [20].

In order to analyse the effect of nutrients in apogamous ferns like *Phegopteris connectilis* our study was carried out at 3% sucrose with varying strength of MS medium and full, none or half concentration of vitamins. The results indicated that full MS medium irrespective of presence of vitamins resulted in significantly high content of Zn, whereas, a significant increase in K, Mg, and Mn content was documented in MS + V. Moreover, during stage-1 increase in biomass and size of gametophytes was observed in treatment 1/2MS + 1/2V. However, a notable increase in number of sporophytes was observed in treatment MS + V. This suggests, that 1/2MS + 1/2V under 50 µmol/m2s, photoperiod:16/8 h, temp. 20–25℃ can be a suitable medium for multiplication of *Phegopteris connectilis* gametophytes. In addition, increase in number of sporophytes in MS + V treatment corresponded to increased content of chlorophyll a (mg/g), chlorophyll b (mg/g), carotenoid (µg/g) and macro- & micro- nutrients namely, K, Mg, and Mn (mg/100 g dry weight).

Green cells of protonemal filament and rhizoids are strongly interdependent, wherein rhizoid growth and development rely entirely on photosynthates supplied from green cells, and further development requires monovalent cations supplied only by rhizoid uptake. These cations stunt growth when present in limiting amount [40]. This is consistent with our findings wherein, significant increase in content of monovalent cation K + has been observed in MS + V, exhibiting highest number of sporophytes. K⁺/ Na⁺ played a vital role in cell expansion in *Onoclea sensiblis* L [40]. Calcium dependent signaling pathway is known for their involvement in plant development (stem elongation and vascular development) and responses to changes in micro-environment in *Diplazium maximum* (D. Don) C. Chr [41]. Furthermore, Ca plays an important role in perception of light signal during germination of *Dryopteris paleacea* Sw [42]. Similarly, Ca and Mn play a crucial role in spore germination in *Osmunda japonica* Thunb [43]. A significant increase in Ca was observed in gametophytes of *Phegopteris connectilis* grown in MS + 1/2V and 1/2MS + 1/2V, with the highest content in MS + V treatment showing an elevated number of sporophytes. In agreement with this, significant increase in Mn content was registered with MS + V. In addition, our results indicated, relatively higher content of Ca and Mn in the presence of vitamins with MS, 1/2MS, 1/4MS, 1/8MS medium.

Since highest number of sporophyte was reported in treatment MS + V at 50 µmol/m^2^s, photoperiod:16/8 h, temp. 20–25℃, it is suitable for induction of *Phegopteris connectilis* sporophytes. Moreover, this demonstrated the role of vitamins in sporophyte induction in this species. Callus growth, embryonic development and somatic growth are directly or indirectly influenced by vitamins along with media or media constituents [44]. Thiamine functionally linked to cytokinin activity, plays a contributory role in the stimulation of callus and root development [45–47]. In addition, biotin and riboflavin both contribute to callus development as well [48, 49]. This is consistent with our findings, in stage-1 wherein MS and 1/2 MS medium with vitamins induced greater number of sporophytes as compared to MS and 1/2 MS medium alone (without vitamins). However, sign of necrosis due to nutrient depletion was observed in 1/4MS and in 1/8MS medium. Treatment with 1/4 MS and 1/8 MS induced stress responses, as demonstrated by visible cellular compaction, increase in rhizoid formation and quantitative decrease in chlorophyll content (Fig no. 10 & 11). Rhizoid formation is a typical response to nutrient depletion as it increases surface area for absorption of nutrients. Camloh et al., [50] mentioned the role of jasmonic acid in rhizoid elongation and division of fern protoplast. Moreover, elongation of rhizoids to meet nutrient requirements can be associated with the jasmonic acid signaling in regulation of gametophyte response to nutrient deficiency [51].

This work intended not only to study effective media composition for sporophyte induction but also to support sporophyte formation and increase (on gametophyte) after in vitro transfer. In vitro cultures of plants offers several advantages like culturing plants in controlled condition, accelerating the production of plants or developing plants to obtain certain metabolites, etc. Therefore, in stage-2, the gametophytes from respective treatments were transferred to MS + V media composition (reference control) as it showed in the highest mean sporophyte induction rate.

As all sporophytes remained viable after transferring, the MS + V (reference control medium) supported not only sporophyte development but also the recovery response of gametophytes exposed to nutrient limitation. Moreover, results suggest the possibility of compensatory growth response of gametophytes in MS medium (stage-1) when transferred onto reference control medium (stage-2). Since the reference control medium (MS + V) contains both full nutrients and vitamins, this response highlights the potential role of vitamins in directing developmental transitions towards sporophyte formation. In addition gametophytes initially cultured on 1/4MS and 1/8MS with and without vitamins also demonstrated similar compensatory growth response wherein stressed phenotype was reverted to a condition characterized by the reappearance of green tissue and reduction of visible necrosis. Compensatory response depends on the type and level of stress limiting growth [52]. In higher plants early-stage environmental stress exhibits compensatory growth after stress relief, driven by hormonal regulation, enhanced nutrient dynamics, and reactivation of photosynthetic and growth-related pathways, enabling partial or complete recovery of growth [53, 54].

Ferns, particularly perennial and winter-hardy species, are frequently exposed to seasonal variations in temperature, light availability, and nutrient supply. The ability of gametophytes to alter growth patterns in response to nutrient availability may indicate an adaptive feature that facilitates resilience under variable environmental conditions. Although the molecular mechanisms governing nutrient perception and signaling in fern gametophytes remain poorly understood compared with those in angiosperms, the observed changes in growth, pigmentation, and sporophyte formation in *P. connectilis* demonstrate a degree of developmental plasticity in response to culture conditions. Such plasticity may have ecological importance by enabling gametophytes to maintain survival and reproductive potential when environmental resources fluctuate. Further studies involving physiological and molecular analyses are required to elucidate the mechanisms underlying nutrient sensing and developmental responses in fern gametophytes. Unlike ferns that rely primarily on sexual reproduction, *P. connectilis* produces sporophytes through apogamy, a reproductive strategy often linked with polyploid lineages in ferns [55]. Although polyploidy may influence physiological traits and resource allocation, there is currently no evidence that apogamous ferns generally possess higher nutrient requirements than sexually reproducing ferns. Nutrient demand is influenced by species-specific ecological adaptations, developmental strategies, and growth characteristics [56, 57]. Compensatory growth following nutrient limitation is extensively studied in angiosperms, where nutrient recovery can stimulate increased biomass production and physiological activity [52–54, 58]. However, comparable responses remain poorly studied in ferns, suggesting that the recovery response observed in *P. connectilis* may provide insights into nutrient-response plasticity in pteridophytes.

## Conclusion

The present study demonstrates that the gametophyte development in *Phegoperis connectilis* was influenced by concentration of mineral salts in the culture medium and vitamin supplementation in combination with the mineral salts enhanced sporophyte formation and supported the recovery of stressed gametophytes, as indicated by the reappearance of green tissue and reduction of visible necrosis. A strong positive correlation between nutrient content and sporophyte production highlights the potential role of nutrient availability in regulating developmental transitions. Although strong associations were observed among nutrient content, physiological parameters, and sporophyte production, these relationships do not establish direct causal relationships and may reflect the combined effects of culture medium composition and associated physiological changes. The use of gametophytes as an experimental system is particularly valuable, as their long-lived nature preserves genetic diversity and provides a stable platform for propagation, even in species where sporophytes are rare or vulnerable. In this context, gametophytes can function analogously to a biological ‘seed bank’ by serving as a reservoir of genetic diversity and propagules, supporting both conservation and large-scale propagation efforts. Our protocol allows for *Phegopteris connectilis* gametophytes to be propagated over multiple cycles in vitro under controlled climatic conditions, and independently of environmental conditions. Moreover, MS + V was identified to be the most effective medium under tested condition for sporophyte induction. The gametophytes appear filamentous and sporophytes can be obtained within 6 weeks of gametophyte culture. Additionally, when considering increase in number of sporophytes formation, gametophytes can be initially grown on MS medium for 10 weeks followed by its transfer to MS + V medium. This resulted in a 2.29-fold increase in sporophyte multiplication. Future studies should focus on the factors regulating gametophyte morphology and their underlying physiological and molecular mechanisms to further refine propagation strategies.

## Supplementary Information

Supplementary Material 1.

## Data Availability

Data will be made available on request.

## References

[1] Driscoll HE, Barrington DS, Gilman AV. A reexamination of the apogamous tetraploid Phegopteris (Thelypteridaceae) from northeastern North America. Rhodora. 2003;105:309–21.

[2] Stroh PA, Humphrey TA, Burkmar RJ, Pescott OL, Roy DB, Walker KJ. *Phegopteris connectilis* (Michx.) Watt. In: BSBI Online Plant Atlas 2020. 2020. Available from: BSBI Online Plant Atlas 2020. https://plantatlas2020.org/atlas/2cd4p9h.x7e. Accessed 14 Jun 2026.

[3] Malati SNLS, Ratna Kumar PK, Rao GMN. Distribution and Density of Pteridophytic flora in Galikonda Hills, 1. Malati SNLS, Ratna Kumar PK, Rao GMN. Distribution and Density of Pteridophytic flora in Galikonda Hills, Eastern Ghats of India, Andhra Pradesh. IOSR Journal Of Pharmacy And Biological Sciences (IOSR-JPBS) e-ISSN. 2022;17:22–7. 10.9790/3008-1703032227.

[4] Nugraheni LS, Survani R, Arindri D. The Inventory of ferns (Pteridophyta) in East Citorek, Lebak, Banten. Al-Hayat. J Biology Appl Biology. 2024;7:71–80. 10.21580/ah.v7i1.20902.

[5] Kholia BS. Indian pteridophytes: a plea for domestication, horticulture, and ex-situ conservation. Indian Fern J. 2021;38:222–64.

[6] Prodanovic D, Biberdzic M, Ciric S, Stanojevic M. Wild growing geophytes in the northern Kosovo and Metohija – potential of ornamental use. Fresenius Environ Bull. 2023;32:2543–55.

[7] Sevier D. A meta-analysis of the human uses of pteridophytic species in Tennessee [honors thesis]. Chattanooga (TN): University of Tennessee at Chattanooga; 2020. Available from: https://scholar.utc.edu/honors-theses/286/.

[8] Pliszko A. Red list of vascular plants of the Western Suwałki Lakeland, north-eastern Poland. Acta Musei Silesiae Sci Naturales. 2017;66:65–73. 10.1515/cszma-2017-0007.

[9] Soare LC, Vişoiu E, Andrei M. Researches concerning the in vitro differentiation of the fern *Phegopteris connectilis* (Michx.) Watt. Notulae Botanicae Horti Agrobotanici Cluj-Napoca. 2007;35(1):7–14. 10.15835/nbha351231.

[10] Grusz AL. A current perspective on apomixis in ferns. J Syst Evol. 2016;54:656–65. 10.1111/jse.12228.

[11] Martin KP, Sini S, Zhang CL, Slater A, Madhusoodanan PV. Efficient induction of apospory and apogamy in vitro in silver fern (*Pityrogramma calomelanos* L). Plant Cell Rep. 2006;25:1300–7. 10.1007/s00299-006-0215-5. 16874526

[12] Ravi BX, Varuvel GVA, Kilimas R, Robert J. Apogamous sporophyte development through spore reproduction of a South Asia’s critically endangered fern: *Pteris tripartita* Sw. Asian Pac J Reprod. 2015;4(2):135–9. 10.1016/S2305-0500(15)30010-5.

[13] Soare LC, Vișoiu E, Dobrescu CM. *In vitro* culture and regeneration of the fern *Dryopteris affinis*, species growing in a protected area. Romanian Biotechnol Lett. 2010;15(1 Suppl):45–54. (rombio.unibuc.ro).

[14] Cordle AR, Irish EE, Cheng C. Apogamy Induction in *Ceratopteris richardii*. Int J Plant Sci. 2007;168. 10.1086/511049.

[15] Whittier DP. The effect of sucrose on apogamy in *Cyrtomium falcatum* Presl. Am Fern J. 1964;54:20–5. 10.2307/1546272.

[16] Podio M, Cáceres ME, Samoluk SS, Seijo JG, Pessino SC, Ortiz JPA, et al. A methylation status analysis of the apomixis-specific region in Paspalum spp. suggests an epigenetic control of parthenogenesis. J Exp Bot. 2014;65:6411–24. 10.1093/jxb/eru354. 25180110

[17] Ferreira de Carvalho J, Oplaat C, Pappas N, Derks M, de Ridder D, Verhoeven KJF. Heritable gene expression differences between apomictic clone members in *Taraxacum officinale*: Insights into early stages of evolutionary divergence in asexual plants. BMC Genomics. 2016;17. 10.1186/s12864-016-2524-6. PMC4782324 26956152

[18] Verhoeven KJF, Van Dijk PJ, Biere A. Changes in genomic methylation patterns during the formation of triploid asexual dandelion lineages. Mol Ecol. 2010;19:315–24. 10.1111/j.1365-294X.2009.04460.x. 20015141

[19] Brown EDW. Apogamy in *Phegopteris polypodioides*. Bull Torrey Bot Club. 1923;50. 10.2307/2479976.

[20] Singh AP, Johari D. *In vitro* studies on gametophyte and gametangia development in homosporous fern—*Actiniopteris radiata*. Plant Cell Tissue Organ Culture. 2025;161. 10.1007/s11240-025-03046-x.

[21] Cordle AR, Irish EE, Cheng CL. Gene expression associated with apogamy commitment in *Ceratopteris richardii*. Sex Plant Reprod. 2012;25:293–304. 10.1007/s00497-012-0198-z. 23007856

[22] Fernández H, Bertrand AM, Sánchez-Tamés R. Influence of tissue culture conditions on apogamy in *Dryopteris affinis* sp. affinis. Planr Cell Tissue Organ Cult. 1996;45:93–7. 10.1007/BF00043434.

[23] Rigby SJ. Induction of apogamy in *Pellaea glabella* var. occidentalis. Am Fern J. 1973;63:158–63. 10.2307/1546796.

[24] Soare LC. *In vitro* development of gametophyte and sporophyte in several fern species. Notulae Botanicae Horti Agrobotanici Cluj-Napoca. 2008;36(1):13–9. 10.15835/nbha36183.

[25] Avila-Pérez MCR, White-Olascoaga L, Arzate-Fernández AM. *vitro* regeneration of leatherleaf fern *Rumohra adiantiformis* (G. Forst.) Ching. Am Fern J. 2011;101(1):25–35. 10.1640/0002-8444-101.1.25.

[26] Cho JS, Jang BK, Park K, Lee HM, Lee CH. Gametophyte propagation and sporophyte formation of Asian chain fern *Woodwardia japonica* (L. f.) Sm. under various medium conditions in vitro and ex vitro. Korean J Plant Resour. 2019;32(6):735–42. 10.7732/kjpr.2019.32.6.735.

[27] Jang BK, Cho JS, Park K, Lee CH. Practical methodology for gametophyte proliferation and sporophyte production in green penny fern (*Lemmaphyllum microphyllum* C. Presl) using mechanical fragmentation. Vitro Cell Dev Biology - Plant. 2020;56:318–24. 10.1007/s11627-020-10055-2.

[28] Tomiczak K, Makowski D, Sliwinska E, Mikuła A. The development of an *in vitro* propagation and conservation system for the endangered serpentine fern *Asplenium cuneifolium* Viv. Plant Cell Tissue Organ Cult. 2023;154:161–75. 10.1007/s11240-023-02524-4.

[29] Murashige T, Skoog F. A revised medium for rapid growth and bioassays with tobacco tissue cultures. Physiol Plant. 1962;15(3):473–97. 10.1111/j.1399-3054.1962.tb08052.x.

[30] Schneider CA, Rasband WS, Eliceiri KW. NIH Image to ImageJ: 25 years of image analysis. Nat Methods. 2012;9:671–5. 10.1038/nmeth.2089. PMC5554542 22930834

[31] Porra RJ. The chequered history of the development and use of simultaneous equations for the accurate determination of chlorophylls a and b. Photosynth Res. 2002;73(1–3):149–56. 10.1023/A:1020470224740. 16245116

[32] Lichtenthaler HK, Wellburn AR. Determinations of total carotenoids and chlorophylls a and b of leaf extracts in different solvents. Biochem Soc Trans. 1983;11(5):591–2. 10.1042/bst0110591.

[33] Farrar DR, Wagner WH. The gametophyte of *Trichomanes holopterum* Kunze. Bot Gaz. 1968;129(3):210–9. 10.1086/336468.

[34] Makgomol K, Sheffield E. Gametophyte morphology and ultrastructure of the extremely deep shade fern, *Trichomanes speciosum*. New Phytol. 2001;151:243–55. 10.1046/j.1469-8137.2001.00160.x. 33873378

[35] Galtier J. The origins and early evolution of the megaphyllous leaf. Int J Plant Sci. 2010;171:641–61. 10.1086/653130.

[36] Marchal M. Quelques remarques sur l’ontogénie des plantules épiphylles de Fougères. Bull de la Societe Botanique de France. 1968;115:223–35. 10.1080/00378941.1968.10838610.

[37] Whittier DP, Steeves TA. Further studies on induced apogamy in ferns. Can J Bot. 1962;40(11):1525–31. 10.1139/b62-148.

[38] Elmore HW, Whittier DP. The role of ethylene in the induction of apogamous buds in Pteridium gametophytes. Planta. 1973;111(1):85–90. 10.1007/BF00386738. 24469421

[39] Kaźmierczak A. Gibberellic acid and ethylene control male sex determination and development of *Anemia phyllitidis* gametophytes. Working with Ferns: Issues and Applications. Springer New York; 2010. pp. 49–65. 10.1007/978-1-4419-7162-3_5.

[40] Racusen RH. Early development in fern gametophytes: Interpreting the transition to prothallial architecture in terms of coordinated photosynthate production and osmotic ion uptake. Ann Botany. 2002;89:227–40. 10.1093/aob/mcf032. PMC4233796 12099354

[41] Sareen B, Thapa P, Joshi R, Bhattacharya A. Proteome Analysis of the Gametophytes of a Western Himalayan Fern *Diplazium maximum* Reveals Their Adaptive Responses to Changes in Their Micro-Environment. Front Plant Sci. 2019;10. 10.3389/fpls.2019.01623. PMC6928197 31921265

[42] Durr S, Scheuerlein R. Characterization of a calcium-requiring phase during phytochrome-mediated fern spore germination of *Dryopteris paleacea* Sw. Planta. 1990;180(3):331–7. 10.1007/BF00192744.

[43] Kamachi H, Noguchi M, Inoue H. Possible involvement of Ca²⁺-dependent protein kinases in spore germination of the fern *Osmunda japonica*. J Plant Biology. 2004;47(1):27–32. 10.1007/BF03030224.

[44] Abrahamian P, Kantharajah A. Effect of Vitamins on in-vitro Organogenesis of Plant. Am J Plant Sci. 2011;02:669–74. 10.4236/ajps.2011.25080.

[45] Barwale UB, Kerns HR, Widholm JM. Plant regeneration from callus cultures of several soybean genotypes via embryogenesis and organogenesis. Planta. 1986;167(4):473–81. 10.1007/BF00391223. 24240363

[46] Tiwari R, Singh AK, Rajam MV. Improved and reliable plant regeneration and Agrobacterium-mediated genetic transformation in soybean (*Glycine max* L). J Crop Sci Biotechnol. 2023;26:275–84. 10.1007/s12892-022-00179-9.

[47] Vollmer R, Espirilla J, Sánchez JC, Arroyo L, Acosta M, Flores G, et al. Thiamine improves in vitro propagation of sweetpotato [*Ipomoea batatas* (L.) Lam.] – confirmed with a wide range of genotypes. Plant Cell Tissue Organ Cult. 2023;152:253–66. 10.1007/s11240-022-02400-7.

[48] Al-Khayri JM. Optimization of biotin and thiamine requirements for somatic embryogenesis of date palm (Phoenix dactylifera L). Vitro Cell Dev Biology. 2001. 10.1079/IVP2001200.

[49] Hesami M, Naderi R, Tohidfar M. Introducing a hybrid artificial intelligence method for high-throughput modeling and optimizing plant tissue culture processes: the establishment of a new embryogenesis medium for chrysanthemum, as a case study. Appl Microbiol Biotechnol. 2020. 10.1007/s00253-020. 33119796

[50] Camloh M, Ravnikar M, Žel J. Jasmonic acid promotes division of fern protoplasts, elongation of rhizoids and early development of gametophytes. Plant Growth Regul. 1996;18(2):121–6. 10.1007/BF00024306.

[51] Shikha D, Jakhar P, Satbhai SB. Role of jasmonate signaling in the regulation of plant responses to nutrient deficiency. J Exp Bot. 2023;74:1221–43. 10.1093/jxb/erac387. 36169640

[52] Oesterheld M, Mcnaughton SJ. Effect of stress and time for recovery on the amount of compensatory growth after grazing. Oecologia. 1991;85(3):305–13. 10.1007/BF00320604. 28312033

[53] Wang H, Zhong L, Fu X, Huang S, Fu H, Shi X, et al. Physiological and Transcriptomic Analyses Reveal the Mechanisms of Compensatory Growth Ability for Early Rice after Low Temperature and Weak Light Stress. Plants. 2022;11. 10.3390/plants11192523. PMC9570567 36235390

[54] Wang XL, Duan PL, Yang SJ, Liu YH, Qi L, Shi J, et al. Corn compensatory growth upon post-drought rewatering based on the effects of rhizosphere soil nitrification on cytokinin. Agric Water Manage. 2020;241. 10.1016/j.agwat.2020.106436.

[55] Grusz AL. A current perspective on apomixis in ferns. Plant Syst Evol. 2016;302:1–10. 10.1111/jse.12228.

[56] Page CN. Ecological strategies in fern evolution: a neopteridological overview. Rev Palaeobot Palynol. 2002;119(1–2):1–33. 10.1016/S0034-6667(01)00127-0.

[57] Soltis PS, Marchant DB, Van de Peer Y, Soltis DE. Polyploidy and genome evolution in plants. Curr Opin Genet Dev. 2015;35:119–25. 10.1016/j.gde.2015.11.003. 26656231

[58] Kudoyarova GR, Dodd IC, Veselov DS, Rothwell SA, Veselov SY. Common and specific responses to availability of mineral nutrients and water. J Exp Bot. 2015;66(8):2133–44. 10.1093/jxb/erv017. PMC4986719 25697793

