# Supplementary material for: In vitro propagation and morphological responses of a winter-hardy fern (Phegopteris connectilis (Michx.) Watt) under varying strength of MS medium and vitamin supplementation
Source: BMC Plant Biol. 2026 Jul 28;26:1822. doi: 10.1186/s12870-026-09611-6 (PMC13693008; doi:10.1186/s12870-026-09611-6)
Supplement: Supplementary file 1 [file 12870_2026_9611_MOESM1_ESM.odt]

 Treatments
Week 6 
Week 7
Week 8
Week 9
Week 10
MS
2ab±1.87
4ab±4.26
5ab±4.79
7abc±4.21
14ab±9.55
MS+V
4a±1.14
10a±7.01
13a±8.92
13a±8.97
17a±12.33
MS+1/2V
4a±2.44
7ab±6.22
12ab±7.56
13a±7.56
16a±7.98
1/2MS
1.4ab±2.6
4ab±3.6
5ab±8.7
5abc±1.22
6ab±8.61
1/2MS+V
3ab±2.7
6ab±6.58
7ab±7.43
8abc±4.82
9ab±7.32
1/2MS+1/2V
3ab±2.38
7ab±1.64
7ab±9.73
10ab±3.11
11ab±14.21
1/4MS
0b±0
0b±0
0b±0
0.2c±0.44
0b±0.44
1/4MS+V
0b±0
0b±0
0b±0
1.4bc±3.13
2ab±3.57
1/4MS+1/2V
0b±0
0b±0
0b±0
0c±0
0b±0
1/8MS
0b±0
0b±0
0.2b±0.44
0.2c±0.44
0b±0.44
1/8MS+V
0b±0
0b±0
0b±0
0c±0
0b±0
1/8MS+1/2V
0b±0
0b±0
0b±0
0c±0
0b±0
S1. Average number of sporophytes in stage-1
 Treatments
Week 1
Week 2
Week 3
Week 4
MS
8bc±2.51
12bc±1.15
18ab±4.93
19ab±4.04
MS+V
18a±2.64
24a±4.35
25a±4.35
25a±4.93
MS+1/2V
15ab±1.52
16ab±2.64
17abc±3.46
18ab±2.3
1/2MS
0c±0
0d±0
5bcd±9.23
6bc±10.39
1/2MS+V
15ab±6.65
17ab±6.02
11abcd±11.15
11bc±6.08
1/2MS+1/2V
6c±4.61
7bcd±7.02
6bcd±5.5
6bc±3.78
1/4MS
0c±0
0d±0
0d±0.57
0.3c±0.57
1/4MS+V
3c±4.16
3cd±4.93
3bcd±4.93
4c±5.29
1/4MS+1/2V
0.6c±0.57
1d±1
1d±1
1c±1
1/8MS
0c±0
2cd±2.88
2cd±2.88
2c±3.46
1/8MS+V
0c±0
0d±0
0d±0
0c±0
1/8MS+1/2V
0c±0
1d±1.73
1d±1.73
1c±1.73
Values are presented as mean ± standard deviation (SD) of biological replicates. The data show the mean of 5 replicates. Treatments followed by the same letter did not differ significantly according to Tukey’s (p=0.05) of ANOVA. MS – Murashige and Skoog medium; V – vitamins 
S2. Average number of sporophytes in stage 2
Values are presented as mean ± standard deviation (SD) of biological replicates. The data show the mean of 3 replicates. Treatments followed by the same letter did not differ significantly according to Tukey’s (p=0.05) of ANOVA. MS – Murashige and Skoog medium; V – vitamins
